# Supplementary material for: Type and amount of help as predictors for impression of helpers
Source: PLoS One. 2020 Dec 11;15(12):e0243808. doi: 10.1371/journal.pone.0243808 (PMC7732071; doi:10.1371/journal.pone.0243808)

# Online supplementary material (OSM) 1. English translations of all conditions of all vignettes included in Study 1a.

## Identified victims vignette

### High amount – Gives to homeless and to shelter

Felicia lives in an average sized town in southern Sweden. She works at a hotel, has no problem regarding her finances and is content with her life. There are some homeless people in the city Felicia lives in.

Felicia usually gives some spare change or a cup of coffee to the homeless when asked. Felicia also donates 80 dollars a month to support a homeless shelter via an automatic payment to help the city’s deprived and homeless.

### Low amount – Gives to homeless and to shelter

Felicia lives in an average sized town in southern Sweden. She works at a hotel, has no problem regarding her finances and is content with her life. There are some homeless people in the city Felicia lives in.

Felicia usually gives some spare change or a cup of coffee to the homeless when asked. Felicia also donates 10 dollars a month to support a homeless shelter via an automatic payment to help the city’s deprived and homeless.

### High amount – Gives only to shelter

Felicia lives in an average sized town in southern Sweden. She works at a hotel, has no problem regarding her finances and is content with her life. There are some homeless people in the city Felicia lives in.

Felicia by principle never gives the homeless any kind of direct aid, even if they are just asking for a cup of coffee. Instead, Felicia donates 80 dollars a month to support a homeless shelter via an automatic payment to help the city’s deprived and homeless.

### Low amount – Gives only to shelter

Felicia lives in an average sized town in southern Sweden. She works at a hotel, has no problem regarding her finances and is content with her life. There are some homeless people in the city Felicia lives in.

Felicia by principle never gives the homeless any kind of direct aid, even if they are just asking for a cup of coffee. Instead, Felicia donates 10 dollars a month to support a homeless shelter via an automatic payment to help the city’s deprived and homeless.

## Emotional reactions vignette

### High amount – Emotionally touched

Aron is 31 years old, single, and works at an insurance company. Aron earns about 2 500 dollars each month and lives in an apartment. Aron is by no means rich, but he also does not have any financial problems.

About a week ago, Aron saw a documentary which described the famine in east Africa. The documentary followed two orphans who to no avail scavenged for food in waste containers.
When Aron saw the documentary he become very emotionally moved. Aron experienced strong compassion towards the people affected by the famine and realized they need help.

After having watched the documentary, Aron decided to set up an automatic payment which donates 30 dollars each month to organizations who help children in poor countries.

### Low amount – Emotionally touched

Aron is 31 years old, single, and works at an insurance company. Aron earns about 2 500 dollars each month and lives in an apartment. Aron is by no means rich, but he also does not have any financial problems.

About a week ago, Aron saw a documentary which described the famine in east Africa. The documentary followed two orphans who to no avail scavenged for food in waste containers.
When Aron saw the documentary he become very emotionally moved. Aron experienced strong compassion towards the people affected by the famine and realized they need help.

After having watched the documentary, Aron decided to set up an automatic payment which donates 6 dollars each month to organizations who help children in poor countries.

### High amount – Not emotionally touched

Aron is 31 years old, single, and works at an insurance company. Aron earns about 2 500 dollars each month and lives in an apartment. Aron is by no means rich, but he also does not have any financial problems.

About a week ago, Aron saw a documentary which described the famine in east Africa. The documentary followed two orphans who to no avail scavenged for food in waste containers.
When Aron saw the documentary, he did not feel very emotionally moved. Aron did not experience any special feelings towards the people affected by the famine, but rationally realized that those affected by the famine need help.

After having watched the documentary, Aron decided to set up an automatic payment which donates 30 dollars each month to organizations who help children in poor countries.

### Low amount – Not emotionally touched

Aron is 31 years old, single, and works at an insurance company. Aron earns about 2 500 dollars each month and lives in an apartment. Aron is by no means rich, but he also does not have any financial problems.

About a week ago, Aron saw a documentary which described the famine in east Africa. The documentary followed two orphans who to no avail scavenged for food in waste containers.
When Aron saw the documentary, he did not feel very emotionally moved. Aron did not experience any special feelings towards the people affected by the famine, but rationally realized that those affected by the famine need help.

After having watched the documentary, Aron decided to set up an automatic payment which donates 6 dollars each month to organizations who help children in poor countries.

## Directness vignette

### High amount – Volunteers at refugee camp

Dan is 46 years old, single and a renowned surgeon and business owner. Dan also has experience of being a military surgeon and is therefore used to serious and stressful situations. For many years, Dan worked as a surgeon at his own very exclusive private clinic in Sweden where he earned over 9 000 dollars a month.

A year ago, Dan felt that he wanted to help the poor people in the world. Dan therefore sold his private clinic and now periodically lives in a refugee camp in Kongo where he volunteers as a doctor.

It has been estimated that because of Dan’s special competences, 60 more lives can be saved each year than was possible before.

### Low amount – Volunteers at refugee camp

Dan is 46 years old, single and a renowned surgeon and business owner. Dan also has experience of being a military surgeon and is therefore used to serious and stressful situations. For many years, Dan worked as a surgeon at his own very exclusive private clinic in Sweden where he earned over 9 000 dollars a month.

A year ago, Dan felt that he wanted to help the poor people in the world. Dan therefore sold his private clinic and now periodically lives in a refugee camp in Kongo where he volunteers as a doctor.

It has been estimated that because of Dan’s special competences, 15 more lives can be saved each year than was possible before.

### High amount – Donates part of salary

Dan is 46 years old, single and a renowned surgeon and business owner. Dan also has experience of being a military surgeon and is therefore used to serious and stressful situations. For many years, Dan worked as a surgeon at his own very exclusive private clinic in Sweden where he earned over 9 000 dollars a month.

A year ago, Dan felt that he wanted to help the poor people in the world. Dan therefore used a part of his salary to fund a hedge fund which every year pays for medical procedures in different refugee camps.

It has been estimated that because of Dan’s hedge fund, 60 more lives can be saved each year than was possible before.

### Low amount – Donates part of salary

Dan is 46 years old, single and a renowned surgeon and business owner. Dan also has experience of being a military surgeon and is therefore used to serious and stressful situations. For many years, Dan worked as a surgeon at his own very exclusive private clinic in Sweden where he earned over 9 000 dollars a month.

A year ago, Dan felt that he wanted to help the poor people in the world. Dan therefore used a part of his salary to fund a hedge fund which every year pays for medical procedures in different refugee camps.

It has been estimated that because of Dan’s hedge fund, 15 more lives can be saved each year than was possible before.

## Personal Sacrifice vignette

### High amount – Experience hardships

Beata is a qualified medical doctor who also speaks fluent Portuguese. During her first years as a medical doctor, Beata worked at a small hospital in Sweden, but she later moved to Rio de Janeiro to work at a small clinic in one of the poorest areas in the city, where there had not until then been any qualified medical doctor present.

Beata works 34 hours a week at the clinic. When Beata is not working, she spends most of her time in the slums trying to experience the same things the poor do there.

It has been estimated that because Beata works at the clinic, 55 more lives can be saved each year than was possible before.

### Low amount – Experience hardships

Beata is a qualified medical doctor who also speaks fluent Portuguese. During her first years as a medical doctor, Beata worked at a small hospital in Sweden, but she later moved to Rio de Janeiro to work at a small clinic in one of the poorest areas in the city, where there had not until then been any qualified medical doctor present.

Beata works 34 hours a week at the clinic. When Beata is not working, she spends most of her time in the slums trying to experience the same things the poor do there.

It has been estimated that because Beata works at the clinic, 11 more lives can be saved each year than was possible before.

### High amount – Enjoy life

Beata is a qualified medical doctor who also speaks fluent Portuguese. During her first years as a medical doctor, Beata worked at a small hospital in Sweden, but she later moved to Rio de Janeiro to work at a small clinic in one of the poorest areas in the city, where there had not until then been any qualified medical doctor present.

Beata works 34 hours a week at the clinic. When Beata is not working, she usually stays at one of her relatives who lives in the richer parts of Rio de Janeiro, where she can fully enjoy the nice weather and the delicious food.

It has been estimated that because Beata works at the clinic, 55 more lives can be saved each year than was possible before.

### Low amount – Enjoy life

Beata is a qualified medical doctor who also speaks fluent Portuguese. During her first years as a medical doctor, Beata worked at a small hospital in Sweden, but she later moved to Rio de Janeiro to work at a small clinic in one of the poorest areas in the city, where there had not until then been any qualified medical doctor present.

Beata works 34 hours a week at the clinic. When Beata is not working, she usually stays at one of her relatives who lives in the richer parts of Rio de Janeiro, where she can fully enjoy the nice weather and the delicious food.

It has been estimated that because Beata works at the clinic, 11 more lives can be saved each year than was possible before.

## Keeping help private vignette

### High amount – Puts certificate in drawer

Eva is 58 years old and is employed as head of department at a big department store. Eva is divorced and has two adult children who no longer live at home. Eva earns 3 000 a month and rents an apartment. Eva has some savings which she is planning to use for traveling abroad in the future.

About a week ago, Eva saw a news segment in which a little girl with late-stage cancer was interviewed. The little girl talked about her condition, her dreams and her fear of death.

After watching the news segment, Eva decided to donate money to the Child Cancer Foundation. The day after the news segment, Eva donated 250 dollars. The money is used both for cancer research and treatment of children with cancer.

When Eva donated, she received a certificate from the Child Cancer Foundation in gratitude for the donation. Eva put the certificate deep inside a drawer and did not show it to anyone.

### Low amount – Puts certificate in drawer

Eva is 58 years old and is employed as head of department at a big department store. Eva is divorced and has two adult children who no longer live at home. Eva earns 3 000 a month and rents an apartment. Eva has some savings which she is planning to use for traveling abroad in the future.

About a week ago, Eva saw a news segment in which a little girl with late-stage cancer was interviewed. The little girl talked about her condition, her dreams and her fear of death.

After watching the news segment, Eva decided to donate money to the Child Cancer Foundation. The day after the news segment, Eva donated 50 dollars. The money is used both for cancer research and treatment of children with cancer.

When Eva donated, she received a certificate from the Child Cancer Foundation in gratitude for the donation. Eva put the certificate deep inside a drawer and did not show it to anyone.

### High amount – Hangs certificate on wall

Eva is 58 years old and is employed as head of department at a big department store. Eva is divorced and has two adult children who no longer live at home. Eva earns 3 000 a month and rents an apartment. Eva has some savings which she is planning to use for traveling abroad in the future.

About a week ago, Eva saw a news segment in which a little girl with late-stage cancer was interviewed. The little girl talked about her condition, her dreams and her fear of death.

After watching the news segment, Eva decided to donate money to the Child Cancer Foundation. The day after the news segment, Eva donated 250 dollars. The money is used both for cancer research and treatment of children with cancer.

When Eva donated, she received a certificate from the Child Cancer Foundation in gratitude for the donation. Eva hung this certificate on the wall outside her office.

### Low amount – Hangs certificate on wall

Eva is 58 years old and is employed as head of department at a big department store. Eva is divorced and has two adult children who no longer live at home. Eva earns 3 000 a month and rents an apartment. Eva has some savings which she is planning to use for traveling abroad in the future.

About a week ago, Eva saw a news segment in which a little girl with late-stage cancer was interviewed. The little girl talked about her condition, her dreams and her fear of death.

After watching the news segment, Eva decided to donate money to the Child Cancer Foundation. The day after the news segment, Eva donated 50 dollars. The money is used both for cancer research and treatment of children with cancer.

When Eva donated, she received a certificate from the Child Cancer Foundation in gratitude for the donation. Eva hung this certificate on the wall outside her office.

## Equal helping vignette

### High amount – Gives to all requesters

Hans is single, blue-collar worker who recently won 750 000 dollars in a state-run lottery. When people win big amounts in lotteries, different charitable organizations usually contact the winner to ask if the winner potentially could donate a small part of the sum to their charity. Hans got contacted by 12 different, serious charitable organizations who specialize in different kinds of aid.

Hans agreed to donate to all of the 12 charitable organizations. Every charity received 3500 dollars which in total amounted to 42 000 dollars.

### Low amount – Gives to all requesters

Hans is single, blue-collar worker who recently won 750 000 dollars in a state-run lottery. When people win big amounts in lotteries, different charitable organizations usually contact the winner to ask if the winner potentially could donate a small part of the sum to their charity. Hans got contacted by 12 different, serious charitable organizations who specialize in different kinds of aid.

Hans agreed to donate to all of the 12 charitable organizations. Every charity received 600 dollars which in total amounted to 7200 dollars.

### High amount – Gives to only one requester

Hans is single, blue-collar worker who recently won 750 000 dollars in a state-run lottery. When people win big amounts in lotteries, different charitable organizations usually contact the winner to ask if the winner potentially could donate a small part of the sum to their charity. Hans got contacted by 12 different, serious charitable organizations who specialize in different kinds of aid.

Hans declined donating to 11 of the 12 organizations and only agreed to donate to an organization which primarily helps poor countries develop their infrastructure. The organization received 42 000 dollars in total from Hans.

### High amount – Gives to only one requester

Hans is single, blue-collar worker who recently won 750 000 dollars in a state-run lottery. When people win big amounts in lotteries, different charitable organizations usually contact the winner to ask if the winner potentially could donate a small part of the sum to their charity. Hans got contacted by 12 different, serious charitable organizations who specialize in different kinds of aid.

Hans declined donating to 11 of the 12 organizations and only agreed to donate to an organization which primarily helps poor countries develop their infrastructure. The organization received 7200 dollars in total from Hans.

## High proportion vignette (not reported in the manuscript and rendered no significant effect: see graphical illustration of results below)

### High amount – High proportion

Gusten is a very successful businessman and multi-millionaire. Gusten is also somewhat of a philanthropist and believes that he who is very fortunate should help the sick and poor. During the recent years, Gusten has chosen to invest 75 million dollars into developing a vaccine against a special kind of blood disease which primarily afflicts children in south-east Asia.

Today, about 375 children die each year to this blood disease. The vaccine is estimated to be 100 % effective and therefore be able to save about 375 lives per year.

### Low amount – High proportion

Gusten is a very successful businessman and multi-millionaire. Gusten is also somewhat of a philanthropist and believes that he who is very fortunate should help the sick and poor. During the recent years, Gusten has chosen to invest 75 million dollars into developing a vaccine against a special kind of blood disease which primarily afflicts children in south-east Asia.

Today, about 75 children die each year to this blood disease. The vaccine is estimated to be 100 % effective and therefore be able to save about 75 lives per year.

### High amount – Low proportion

Gusten is a very successful businessman and multi-millionaire. Gusten is also somewhat of a philanthropist and believes that he who is very fortunate should help the sick and poor. During the recent years, Gusten has chosen to invest 75 million dollars into developing a vaccine against a special kind of blood disease which primarily afflicts children in south-east Asia.

Today, about 1170 children each year die to this blood disease. The vaccine is estimated to be 30 % effective and therefore be able to save about 375 lives per year.

### Low amount – Low proportion

Gusten is a very successful businessman and multi-millionaire. Gusten is also somewhat of a philanthropist and believes that he who is very fortunate should help the sick and poor. During the recent years, Gusten has chosen to invest 75 million dollars into developing a vaccine against a special kind of blood disease which primarily afflicts children in south-east Asia.

Today, about 250 children each year die to this blood disease. The vaccine is estimated to be 30 % effective and therefore be able to save about 75 lives per year.


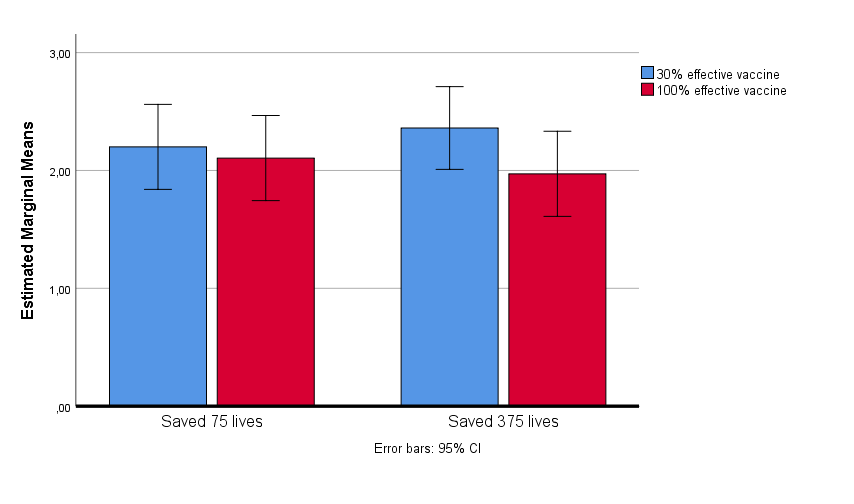

Supplement: S1 File — (DOCX) [file pone.0243808.s001.docx]
