# Supplementary material for: Type and amount of help as predictors for impression of helpers
Source: PLoS One. 2020 Dec 11;15(12):e0243808. doi: 10.1371/journal.pone.0243808 (PMC7732071; doi:10.1371/journal.pone.0243808)
Supplement: S2 File — (DOCX) [file pone.0243808.s002.docx]

# Online supplementary material (OSM) 2. All conditions of all vignettes included in Study 1b.

## Identified victims vignette

### High amount – Gives to homeless and to shelter

Jessica lives in an average sized town in the US. Jessica works at a hotel, has no problem regarding her finances and is content with her life.

There are plenty of homeless people in the city Jessica lives in. Jessica is being asked for money several times a day and often gives some spare change or a cup of coffee to the homeless when asked.

Jessica also donates 100 dollars a month to a small local shelter that efficiently helps the homeless with food, temporary lodging and social support.

### Low amount – Gives to homeless and to shelter

Jessica lives in an average sized town in the US. Jessica works at a hotel, has no problem regarding her finances and is content with her life.

There are plenty of homeless people in the city Jessica lives in. Jessica is being asked for money several times a day and often gives some spare change or a cup of coffee to the homeless when asked.

Jessica also donates 20 dollars a month to a small local shelter that efficiently helps the homeless in the city with food, temporary lodging and social support.

### High amount – Gives only to shelter

Jessica lives in an average sized town in the US. Jessica works at a hotel, has no problem regarding her finances, and is content with her life.

There are plenty of homeless people in the city Jessica lives in. Jessica is being asked for money several times a day but has decided to never give the homeless any kind of direct aid, not even some spare change or a cup of coffee.

Instead, Jessica donates 100 dollars a month to a small local shelter who efficiently helps the homeless in the city with food, temporary lodging and social support.

### Low amount – Gives only to shelter

Jessica lives in an average sized town in the US. Jessica works at a hotel, has no problem regarding her finances, and is content with her life.

There are plenty of homeless people in the city Jessica lives in. Jessica is being asked for money several times a day but has decided to never give the homeless any kind of direct aid, not even some spare change or a cup of coffee.

Instead, Jessica donates 20 dollars a month to a small local shelter that efficiently helps the homeless in the city with food, temporary lodging and social support.

## Emotional reactions vignette

### High amount – Emotionally touched

Paul is 31 years old, single, and has a full-time job at an insurance company. Paul is by no means rich, but he also does not have any financial problems.

About a week ago, Paul saw a documentary which described the famine in east Africa. The documentary showed two orphans searching for food in waste containers. Paul felt very emotionally moved by the documentary and experienced strong compassion towards the people affected by the famine. Paul realized that those affected by the famine needed help

After watching the documentary, Paul subscribed to a charity program. Fifty dollars is deducted from his bank account each month and sent to a charitable organization that helps African children in need.

### Low amount – Emotionally touched

Paul is 31 years old, single, and has a full-time job at an insurance company. Paul is by no means rich, but he also does not have any financial problems.

About a week ago, Paul saw a documentary which described the famine in east Africa. The documentary showed two orphans searching for food in waste containers. Paul felt very emotionally moved by the documentary, and experienced strong compassion towards the people affected by the famine. Paul realized that those affected by the famine needed help.

After watching the documentary, Paul subscribed to a charity program. Ten dollars is deducted from his bank account each month and sent to a charitable organization that helps African children in need.

### High amount – Not emotionally touched

Paul is 31 years old, single, and has a full-time job at an insurance company. Paul is by no means rich, but he also does not have any financial problems.

About a week ago, Paul saw a documentary which described the famine in east Africa. The documentary showed two orphans searching for food in waste containers. Paul did not feel emotionally moved by the documentary nor did he experience any special feelings towards the people affected by the famine. Still, Paul realized that those affected by the famine needed help.

After watching the documentary, Paul subscribed to a charity program. Fifty dollars is deducted from his bank account each month and sent to a charitable organization that helps African children in need.

### Low amount – Not emotionally touched

Paul is 31 years old, single, and has a full-time job at an insurance company. Paul is by no means rich, but he also does not have any financial problems.

About a week ago, Paul saw a documentary which described the famine in east Africa. The documentary showed two orphans searching for food in waste containers. Paul did not feel emotionally moved by the documentary nor did he experience any special feelings towards the people affected by the famine. Still, Paul realized that those affected by the famine needed help.

After watching the documentary, Paul subscribed to a charity program. Ten dollars is deducted from his bank account each month and sent to a charitable organization that helps African children in need.

## Empathy vignette

### High amount – Feels sorry for the victim

Laura, a 30-year-old office worker arrives home to her three-bedroom apartment and picks up her mail. Apart from the usual bills and advertisements, there’s a letter from a well-known and reputable charity organization.

The organization is asking the recipient of the letter to sign up to a monthly subscription program. The program deducts any amount of money from the subscribers account every month and uses it to help starving people in sub-Saharan Africa.

Laura thinks about signing up to the subscription and realizes that if she does not sign up, people who she could help will continue to suffer. Because she does not want other people to suffer, she decides to sign up. Laura decides to donate 30 dollars every month.

### Low amount – Feels sorry for the victim

Laura, a 30-year-old office worker arrives home to her three-bedroom apartment and picks up her mail. Apart from the usual bills and advertisements, there’s a letter from a well-known and reputable charity organization.

The organization is asking the recipient of the letter to sign up to a monthly subscription program. The program deducts any amount of money from the subscribers account every month and uses it to help starving people in sub-Saharan Africa.

 Laura thinks about signing up to the subscription and realizes that if she does not sign up, people who she could help will continue to suffer. Because she does not want other people to suffer, she decides to sign up. Laura decides to donate 6 dollars every month.

### High amount – Wants to avoid own distress

Laura, a 30-year-old office worker arrives home to her three-bedroom apartment and picks up her mail. Apart from the usual bills and advertisements, there’s a letter from a well-known and reputable charity organization.

The organization is asking the recipient of the letter to sign up to a monthly subscription program. The program deducts any amount of money from the subscribers account every month and uses it to help starving people in sub-Saharan Africa.

Laura thinks about signing up to the subscription and realizes that if she does not sign up, she will feel guilt and perceive herself as a bad person. To avoid feeling bad, she decides to sign up. Laura decides to donate 30 dollars every month.

### Low amount – Wants to avoid own distress

Laura, a 30-year-old office worker arrives home to her three-bedroom apartment and picks up her mail. Apart from the usual bills and advertisements, there’s a letter from a well-known and reputable charity organization.

The organization is asking the recipient of the letter to sign up to a monthly subscription program. The program deducts any amount of money from the subscribers account every month and uses it to help starving people in sub-Saharan Africa.

Laura thinks about signing up to the subscription and realizes that if she does not sign up, she will feel guilt and perceive herself as a bad person. To avoid feeling bad, she decides to sign up. Laura decides to donate 6 dollars every month.

## Directness vignette

### High amount – Volunteers at refugee camp

James is 46 years old, single, and a very skilled surgeon and business owner. James also has experience of being a military surgeon and is therefore used to serious and stressful situations.

For many years, James worked as a surgeon at his own very exclusive private clinic in Hollywood where he earned over 20 000 dollars a month.

A year ago, James felt that he wanted to help people in need. He decided to sell his private clinic and start working as a volunteer for Doctors without Borders. James now works as a voluntary physician at a large refugee camp in Turkey.

Because of James’s voluntary work, about 140 additional lives can be saved each year in the large refugee camp.

### Low amount – Volunteers at refugee camp

James is 46 years old, single, and a very skilled surgeon and business owner. James also has experience of being a military surgeon and is therefore used to serious and stressful situations.

For many years, James worked as a surgeon at his own very exclusive private clinic in Hollywood where he earned over 20 000 dollars a month.

A year ago, James felt that he wanted to help people in need. He decided to sell his private clinic and start working as a volunteer for Doctors without Borders. James now works as a voluntary physician at a large refugee camp in Turkey.

Because of James’s voluntary work, about 28 additional lives can be saved each year in the large refugee camp.

### High amount – Donates part of salary

James is 46 years old, single, and a very skilled surgeon and business owner. James also has experience of being a military surgeon and is therefore used to serious and stressful situations.

For many years, James has been working as a surgeon at his own very exclusive private clinic in Hollywood where he earns over 20 000 dollars a month.

A year ago, James felt that he wanted to help people in need. He started to donate half of his monthly income to Doctors without Borders. James still works at his Hollywood clinic, but his donations pay supplies and medicines as well as salaries to Syrian physicians working in a large refugee camp in Turkey.

Because of James’s monthly donations, about 140 additional lives can be saved each year in the large refugee camp.

### Low amount – Donates part of salary

James is 46 years old, single, and a very skilled surgeon and business owner. James also has experience of being a military surgeon and is therefore used to serious and stressful situations.

For many years, James has been working as a surgeon at his own very exclusive private clinic in Hollywood where he earns over 20 000 dollars a month.

A year ago, James felt that he wanted to help people in need. He started to donate some of his monthly income to Doctors without Borders. James still works at his Hollywood clinic, but his donations pay supplies and medicines as well as salaries to Syrian physicians working in a large refugee camp in Turkey.

Because of James’s monthly donations, about 28 additional lives can be saved each year in the large refugee camp.

## Personal Sacrifice vignette

### High amount – Experience hardships

Linda is a medical doctor who also speaks fluent Portuguese. During her first years as a medical doctor, Linda worked at a small hospital in the US, but she later moved to Rio de Janeiro to work at a small clinic in one of the poorest areas in the city.

Linda works 34 hours a week at the clinic. When she’s not working, she spends most of her time in the slums trying to gain a greater understanding about the hardships people have to go through there.

Before Linda began working at the clinic, there were no other medical doctors working in that area. According to the nurses at the small clinic where Linda works, 40 patients that otherwise would have died, have been saved by Linda.

### Low amount – Experience hardships

Linda is a medical doctor who also speaks fluent Portuguese. During her first years as a medical doctor, Linda worked at a small hospital in the US, but she later moved to Rio de Janeiro to work at a small clinic in one of the poorest areas in the city.

Linda works 34 hours a week at the clinic. When she’s not working, she spends most of her time in the slums trying to gain a greater understanding about the hardships people have to go through there.

Before Linda began working at the clinic, there were two other medical doctors working in that area. According to the nurses at the small clinic where Linda works, 5 patients that otherwise would have died, have been saved by Linda.

### High amount – Enjoys life

Linda is a medical doctor who also speaks fluent Portuguese. During her first years as a medical doctor, Linda worked at a small hospital in the US, but she later moved to Rio de Janeiro to work at a small clinic in one of the poorest areas in the city.

Linda works 34 hours a week at the clinic. When she’s not working, she likes to stay with one of her relatives who lives in the richer parts of Rio de Janeiro, where she can fully enjoy the nice weather, the beach parties, and the delicious Brazilian food.

Before Linda began working at the clinic, there were no other medical doctors working in that area. According to the nurses at the small clinic where Linda works, 40 patients that otherwise would have died, have been saved by Linda.

### Low amount – Enjoys life

Linda is a medical doctor who also speaks fluent Portuguese. During her first years as a medical doctor, Linda worked at a small hospital in the US, but she later moved to Rio de Janeiro to work at a small clinic in one of the poorest areas in the city.

Linda works 34 hours a week at the clinic. When she’s not working, she likes to stay with one of her relatives who lives in the richer parts of Rio de Janeiro, where she can fully enjoy the nice weather, the beach parties and the delicious Brazilian food.

Before Linda began working at the clinic, there were two other medical doctors working in that area. According to the nurses at the small clinic where Linda works, 5 patients that otherwise would have died, have been saved by Linda.

## Keeping help private vignette

### High amount – Puts certificate in drawer

Susan is 58 years old and is employed as senior manager at a big department store. Since she is divorced and has two adult children, she lives alone in her own house which she owns without any mortgage. She also has some savings which she is planning to use for traveling abroad in the future.

A week ago, Susan saw a news segment in which a little girl with late-stage cancer was interviewed. The little girl talked about her condition, her dreams and her fear of death.

After watching the news segment, Susan decided to donate money to the Child Cancer Foundation. The day after the news segment, Susan made a one-time donation of 300 dollars. The money donated is used both for research and treatment of children with cancer.

Because Susan donated money, she was given a certificate from the Child Cancer Foundation. Susan put the certificate in one of her drawers and soon forgot about it.

### Low amount – Puts certificate in drawer

Susan is 58 years old and is employed as senior manager at a big department store. Since she is divorced and has two adult children, she lives alone in her own house which she owns without any mortgage. She also has some savings which she is planning to use for traveling abroad in the future.

A week ago, Susan saw a news segment in which a little girl with late-stage cancer was interviewed. The little girl talked about her condition, her dreams and her fear of death.

After watching the news segment, Susan decided to donate money to the Child Cancer Foundation. The day after the news segment, Susan made a one-time donation of 60 dollars. The money donated is used both for research and treatment of children with cancer.

Because Susan donated money, she was given a certificate from the Child Cancer Foundation. Susan put the certificate in one of her drawers and soon forgot about it.

### High amount – Hangs certificate on wall

Susan is 58 years old and is employed as senior manager at a big department store. Since she is divorced and has two adult children, she lives alone in her own house which she owns without any mortgage. She also has some savings which she is planning to use for traveling abroad in the future.

A week ago, Susan saw a news segment in which a little girl with late-stage cancer was interviewed. The little girl talked about her condition, her dreams and her fear of death.

After watching the news segment, Susan decided to donate money to the Child Cancer Foundation. The day after the news segment, Susan made a one-time donation of 300 dollars. The money donated is used both for research and treatment of children with cancer.

Because Susan donated money, she was given a certificate from the Child Cancer Foundation. Susan hung this certificate on the wall outside her office.

### Low amount – Hangs certificate on wall

Susan is 58 years old and is employed as senior manager at a big department store. Since she is divorced and has two adult children, she lives alone in her own house which she owns without any mortgage. She also has some savings which she is planning to use for traveling abroad in the future.

A week ago, Susan saw a news segment in which a little girl with late-stage cancer was interviewed. The little girl talked about her condition, her dreams and her fear of death.

After watching the news segment, Susan decided to donate money to the Child Cancer Foundation. The day after the news segment, Susan made a one-time donation of 60 dollars. The money donated is used both for research and treatment of children with cancer.

Because Susan donated money, she was given a certificate from the Child Cancer Foundation. Susan hung this certificate on the wall outside her office.

## Non-tainted altruism vignette

### High amount – Altruistic motivation

Kevin is a 22-year-old university student living in Texas. His main interest is video games, but he is also good at bowling.

Recently, Kevin has felt that he should do something for his community. Because of this, he decides to volunteer at a soup kitchen that feeds homeless people.

Kevin does a good job at the soup kitchen. He becomes popular among the homeless and among the other volunteers. He volunteers at the soup kitchen about 10 hours a week.

### Low amount – Altruistic motivation

Kevin is a 22-year-old university student living in Texas. His main interest is video games, but he is also good at bowling.

Recently, Kevin has felt that he should do something for his community. Because of this, he decides to volunteer at a soup kitchen that feeds homeless people.

Kevin does a good job at the soup kitchen. He becomes popular among the homeless and among the other volunteers. He volunteers at the soup kitchen about 2 hours a week.

### High amount – Mixed motivation

Kevin is a 22-year-old university student living in Texas. His main interest is video games, but he is also good at bowling.

Kevin has a secret crush on his classmate Nathalie. Nathalie is dedicated to philanthropy and volunteers at a soup kitchen that feeds homeless people.

To get to know Nathalie better and at the same time do something good for his community, Kevin decides to volunteer at the soup kitchen.

Kevin does a good job at the soup kitchen. He becomes popular among the homeless and among the other volunteers. He volunteers at the soup kitchen about 10 hours a week.

### Low amount – Mixed motivation

Kevin is a 22-year-old university student living in Texas. His main interest is video games, but he is also good at bowling.

Kevin has a secret crush on his classmate Nathalie. Nathalie is dedicated to philanthropy and volunteers at a soup kitchen that feeds homeless people. To get to know Nathalie better and at the same time do something good for his community, Kevin decides to volunteer at the soup kitchen.

Kevin does a good job at the soup kitchen. He becomes popular among the homeless and among the other volunteers. He volunteers at the soup kitchen about 2 hours a week.

## Matching other’s donation vignette

### High amount – Matches acquaintance

Anna and her acquaintance Rebecca are out on a business lunch when they are approached by a volunteer working for a reputable charity organization. The volunteer asks if they could potentially donate a small amount towards helping a local cat shelter.

Rebecca immediately agrees and donates 20 dollars.

Anna notices the amount Rebecca donated and then also donates 20 dollars.

### Low amount – Matches acquaintance

Anna and her acquaintance Rebecca are out on a business lunch when they are approached by a volunteer working for a reputable charity organization. The volunteer asks if they could potentially donate a small amount towards helping a local cat shelter.

Rebecca immediately agrees and donates 4 dollars.

Anna notices the amount Rebecca donated and then also donates 4 dollars.

### High amount – Surpasses acquaintance

Anna and her acquaintance Rebecca are out on a business lunch when they are approached by a volunteer working for a reputable charity organization. The volunteer asks if they could potentially donate a small amount towards helping a local cat shelter.

Rebecca immediately agrees and donates 10 dollars.

Anna notices the amount Rebecca donated and then donates 20 dollars.

### Low amount – Surpasses acquaintance

Anna and her acquaintance Rebecca are out on a business lunch when they are approached by a volunteer working for a reputable charity organization. The volunteer asks if they could potentially donate a small amount towards helping a local cat shelter.

Rebecca immediately agrees and donates 2 dollars.

Anna notices the amount Rebecca donated and then donates 4 dollars.

## Equal helping vignette

### High amount – Gives to all requesters

John is a 45-year-old, single, blue-collar worker. Recently John won 1 million dollars in a state-run lottery. When people win big amounts in lotteries, different charitable organizations routinely contact the winner to ask if he/she potentially could donate a part of the sum to their charity.

John got contacted by 12 different, respected charitable organizations who specialize in different kinds of aid for those in need.

John agreed to donate to all of the 12 charitable organizations who contacted him. He chose to donate equal amounts to each of the organizations.

In total, John donated 30 000 dollars to the charitable organizations.

### Low amount – Gives to all requesters

John is a 45-year-old, single, blue-collar worker. Recently John won 1 million dollars in a state-run lottery. When people win big amounts in lotteries, different charitable organizations routinely contact the winner to ask if he/she potentially could donate a part of the sum to their charity.

John got contacted by 12 different, respected charitable organizations who specialize in different kinds of aid for those in need.

John agreed to donate to all of the 12 charitable organizations who contacted him. He chose to donate equal amounts to each of the organizations.

In total, John donated 5 000 dollars to the charitable organizations.

### High amount – Gives to only one requester

John is a 45-year-old, single, blue-collar worker. Recently John won 1 million dollars in a state-run lottery. When people win big amounts in lotteries, different charitable organizations routinely contact the winner to ask if he/she potentially could donate a part of the sum to their charity.

John got contacted by 12 different, respected charitable organizations who specialize in different kinds of aid for those in need.

John declined to donate to 11 of the 12 charitable organizations who contacted him. He only agreed to donate towards one of the charitable organizations that helps poor countries develop their infrastructure.

In total, John donated 30 000 dollars to that charitable organization.

*Low amount – Gives to only one requester*

John is a 45-year-old, single, blue-collar worker. Recently John won 1 million dollars in a state-run lottery. When people win big amounts in lotteries, different charitable organizations routinely contact the winner to ask if he/she potentially could donate a part of the sum to their charity.

John got contacted by 12 different, respected charitable organizations who specialize in different kinds of aid for those in need.

John declined to donate 11 of the 12 charitable organizations who contacted him. He only agreed to donate towards one of the charitable organizations that helps poor countries develop their infrastructure.

In total, John donated 5 000 dollars to that charitable organization.

***Changing amount vignette***

*High amount – Increases donation*

Robert is 50 years old and works as a dentist. On a Saturday when he is out shopping, he is approached by a volunteer working for a well-known, reputable charity organization.

The volunteer asks if he would like to help poor people get vaccination against Malaria in Africa by subscribing to a program that deducts money from his bank account every month towards this cause. Robert agrees, and he gets a form which he fills in at home and starts donating 40 dollars each month.

After donating towards the cause for 12 months, Robert decides he wants to help some more. He therefore increases the amount he donates from 40 to 50 dollars each month.

*Low amount – Increases donation*

Robert is 50 years old and works as a dentist. On a Saturday when he is out shopping, he is approached by a volunteer working for a well-known, reputable charity organization.

The volunteer asks if he would like to help poor people get vaccination against Malaria in Africa by subscribing to a program that deducts money from his bank account every month towards this cause. Robert agrees, and he gets a form which he fills in at home and starts donating 5 dollars each month.

After donating towards the cause for 12 months, Robert decides he wants to help some more. He therefore increases the amount he donates from 5 to 15 dollars each month.

*High amount – Decreases donation*

Robert is 50 years old and works as a dentist. On a Saturday when he is out shopping, he is approached by a volunteer working for a well-known and reputable charity organization.

The volunteer asks if he would like to help poor people get vaccination against Malaria in Africa by subscribing to a program that deducts money from donors’ bank accounts every month towards this cause. Robert agrees, and he gets a form which he fills in at home and starts donating 60 dollars each month.

After donating towards the cause for 12 months, Robert decides he wants to keep some more money to himself. He therefore decreases the amount he donates from 60 to 50 dollars each month.

*Low amount – Decreases donation*

Robert is 50 years old and works as a dentist. On a Saturday when he is out shopping, he is approached by a volunteer working for a well-known and reputable charity organization.

The volunteer asks if he would like to help poor people get vaccination against Malaria in Africa by subscribing to a program that deducts money from donors’ bank accounts every month towards this cause. Robert agrees, and he gets a form which he fills in at home and starts donating 25 dollars each month.

After donating towards the cause for 12 months, Robert decides he wants to keep some more money to himself. He therefore decreases the amount he donates from 25 to 15 dollars each month.
