# Supplementary material for: Type and amount of help as predictors for impression of helpers
Source: PLoS One. 2020 Dec 11;15(12):e0243808. doi: 10.1371/journal.pone.0243808 (PMC7732071; doi:10.1371/journal.pone.0243808)
Supplement: S3 File — (F-statistics and significance-values). (DOCX) [file pone.0243808.s003.docx]

# Online supplementary material (OSM) 3. Main effects and interaction effects in Study 1.

Table S3: Results from the 2×2 ANOVAS (main effects and interaction effect) from all vignettes included in Study 1a and 1b. The significant interaction effects are briefly explained below

|  | **Type of help main effect** | | **Amount of help main effect** | | **Type*Amount interaction effect** | |
| --- | --- | --- | --- | --- | --- | --- |
| **Vignette** | ***Study 1a*** | ***Study 1b*** | ***Study 1a*** | ***Study 1b*** | ***Study 1a*** | ***Study 1b*** |
| Emotional reactions | *F*[1, 139] = 14.21,  *p* < .001, *η*_p_^2^ = .093*** | *F*[1, 455] = 48.16,  *p* < .001, *η*_p_^2^ = .096*** | *F*[1, 139] = 1.19,  *p* = .276, *η*_p_^2^ = .009 | *F*[1, 455] = 3.15,  *p* = .076, *η*_p_^2^ = .007† | *F*[1, 139] = 0.11,  *p* = .747 | *F*[1, 455] = 0.40,  *p* = .530 |
| Empathy |  | *F*[1, 455] = 90.44,  *p* < .001, *η*_p_^2^ = .166*** |  | *F*[1, 455] = 4.22,  *p* = .041, *η*_p_^2^ = .009* |  | *F*[1, 455] = 0.94,  *p* = .759 |
| Non-tainted altruism |  | *F*[1, 455] = 163.77,  *p* < .001, *η*_p_^2^ = .265*** |  | *F*[1, 455] = 16.73,  *p* < .001, *η*_p_^2^ = .035*** |  | *F*[1, 455] = 1.51,  *p* = .219 |
| Identified victims | *F*[1, 137] = 12.24,  *p* = .001, *η*_p_^2^ = .082** | *F*[1, 455] = 39.08,  *p* < .001, *η*_p_^2^ = .079*** | *F*[1, 137] = 0.63,  *p* = .429, *η*_p_^2^ = .005 | *F*[1, 455] = 10.41,  *p* = .001, *η*_p_^2^ = .022** | *F*[1,137] = 2.16,  *p* = .144 | *F*[1, 455] = 0.29,  *p* = .593. |
| Directness | *F*[1, 136] = 3.92,  *p* = .050, *η*_p_^2^ = .028* | *F*[1, 455] = 3.00,  *p* = .084, *η*_p_^2^ = .007† | *F*[1, 136] = 6.25,  *p* = .014, *η*_p_^2^ = .044* | *F*[1, 455] = 2.77,  *p* = .097, *η*_p_^2^ = .006† | *F*[1, 136] = 1.09,  *p* = .299 | *F*[1, 455] = 8.06,  *p* = .005** |
| Personal sacrifice | *F*[1, 139] = 5.59,  *p* = .019, *η*_p_^2^ = .039* | *F*[1, 455] = 65.80,  *p* < .001, *η*_p_^2^ = .126*** | *F*[1, 139] = 3.44,  *p* = .066, *η*_p_^2^ = .024† | *F*[1, 455] = 7.73,  *p* = .006, *η*_p_^2^ = .017** | *F*[1, 139] = 0.19,  *p* = .665 | *F*[1, 455] = 4.04,  *p* = .045* |
| Keeping help private | *F*[1, 137] = 9.25,  *p* = .003, *η*_p_^2^ = .063** | *F*[1, 455] = 0.08,  *p* = .774, *η*_p_^2^ = 0 | *F*[1, 137] = 1.99,  *p* = .160, *η*_p_^2^ = .014 | *F*[1, 455] = 2.87,  *p* = .091, *η*_p_^2^ = .006† | *F*[1, 137] = 0.67,  *p* = .415 | *F*[1, 455] = 1.45,  *p* = .229. |
| Matching others |  | *F*[1, 455] = 6.55,  *p* = .011, *η*_p_^2^ = .014* |  | *F*[1, 455] = 8.96,  *p* = .003, *η*_p_^2^ = .019** |  | *F*[1, 455] = 5.16,  *p* = .024* |
| Equal helping | *F*[1, 136] = 6.01,  *p* = .016, *η*_p_^2^ = .042* | *F*[1, 455] = 56.47,  *p* < .001, *η*_p_^2^ = .110*** | *F*[1, 136] = 6.80,  *p* = .010, *η*_p_^2^ = .048* | *F*[1, 455] = 19.45,  *p* < .001, *η*_p_^2^ = .041*** | *F*[1, 136] = 1.10,  *p* = .296 | *F*[1, 455] = 1.69,  *p* = .194. |
| Changing amount |  | *F*[1, 455] = 56.54,  *p* < .001, *η*_p_^2^ = .111*** |  | *F*[1, 455] = 1.85,  *p* = .174, *η*_p_^2^ = .004 |  | *F*[1, 455] = 0.71,  *p* = .707. |
| High proportion (not included in manuscript but see S1) | *F*[1, 138] = 1.78,  *p* = .184, *η*_p_^2^ = .013 |  | *F*[1, 138] = 0.01,  *p* = .941, *η*_p_^2^ = 0 |  | *F*[1, 138] = 0.66,  *p* = .420 |  |

*** = p <.001, ** = p < .01, * = p < .05, † = p < .1

## Directness vignette

The interaction effect size was *η*_p_^2^ = .02 [.00 − .04]
This interaction can be understood as the amount of helping positively influencing impressions when helping is provided indirectly but not when helping is provided directly.

## Personal sacrifice vignette

The interaction effect size was *η*_p_^2^ = .01 [.00 − .03]. This interaction can be understood as the amount of helping positively influencing impressions when the helper enjoyed a luxurious lifestyle but not when she experienced hardships.

## Matching others vignette

The interaction effect size was *η*_p_^2^ = .01 [.00 − .03] This interaction can be understood as the amount of help positively influencing impressions when the helper is matching another person’s donation but not when she is surpassing another person’s donation.
