# Supplementary material for: Type and amount of help as predictors for impression of helpers
Source: PLoS One. 2020 Dec 11;15(12):e0243808. doi: 10.1371/journal.pone.0243808 (PMC7732071; doi:10.1371/journal.pone.0243808)
Supplement: S4 File — (DOCX) [file pone.0243808.s004.docx]

# Online supplementary material (OSM) 4. English translations of all conditions of all vignettes included in Study 2.

## Directness vignette Individual

### High amount – Helps directly

James is 46 years old, single, and a very skilled surgeon and business owner. James also has experience of being a military surgeon and is therefore used to serious and stressful situations.

For many years, James worked as a plastic surgeon at his own very exclusive private clinic in Hollywood where he earned over 20 000 dollars a month.

A year ago, James felt that he wanted to help people in need. Therefore he decided to sell his private clinic and start working for Doctors without Borders. James now works as a surgeon at a large refugee camp in Turkey and does not earn more than the local doctors.

Because of James’s voluntary work, about 140 additional lives can be saved each year in the refugee camp.

### Low amount – Helps directly

James is 46 years old, single, and a very skilled surgeon and business owner. James also has experience of being a military surgeon and is therefore used to serious and stressful situations.

For many years, James worked as a plastic surgeon at his own very exclusive private clinic in Hollywood where he earned over 20 000 dollars a month.

A year ago, James felt that he wanted to help people in need. Therefore he decided to sell his private clinic and start working for Doctors without Borders. James now works as a surgeon at a large refugee camp in Turkey and does not earn more than the local doctors.

Because of James’s voluntary work, about 28 additional lives can be saved each year in the refugee camp.

### High amount – Helps indirectly

James is 46 years old, single, and a very skilled surgeon and business owner. James also has experience of being a military surgeon and is therefore used to serious and stressful situations.

For many years, James worked as a plastic surgeon at his own very exclusive private clinic in Hollywood where he earned over 20 000 dollars a month.

A year ago, James felt that he wanted to help people in need. Therefore, he started to donate 50 % of his monthly income to the charity organization Doctors without Borders. James still works at his Hollywood clinic, but his donations pay supplies and medicines as well as salaries to Syrian physicians working at a large refugee camp in Turkey.

Because of James’s donations, about 140 additional lives can be saved each year in the refugee camp.

### Low amount – Helps indirectly

James is 46 years old, single, and a very skilled surgeon and business owner. James also has experience of being a military surgeon and is therefore used to serious and stressful situations.

For many years, James worked as a plastic surgeon at his own very exclusive private clinic in Hollywood where he earned over 20 000 dollars a month.

A year ago, James felt that he wanted to help people in need. Therefore, he started to donate 50 % of his monthly income to the charity organization Doctors without Borders. James still works at his Hollywood clinic, but his donations pay supplies and medicines as well as salaries to Syrian physicians working at a large refugee camp in Turkey.

Because of James’s donations, about 28 additional lives can be saved each year in the refugee camp.

## Directness vignette Corporation

### High amount – Helps directly

The pharmaceutical company Juncos have specialized in manufacturing an effective libido-increasing supplement. Many talented scientists work at Juncos, and for many years they have profited over 5 million dollars yearly.

Recently, the chair members at Juncos, with the support of the stakeholders, decided that the company should strive to not only profit but also help others. Therefore the company stopped producing the very profitable supplement and instead started manufacturing cheap medicines and vaccines which are needed in the world’s poorest countries.

Juncos profit is estimated to decrease by a lot in the near future, but according to the United Nations about 1400 more lives are saved yearly because Juncos changed their goals.

### Low amount – Helps directly

The pharmaceutical company Juncos have specialized in manufacturing an effective libido-increasing supplement. Many talented scientists work at Juncos, and for many years they have profited over 5 million dollars yearly.

Recently, the chair members at Juncos, with the support of the stakeholders, decided that the company should strive to not only profit but also help others. Therefore the company stopped producing the very profitable supplement and instead started manufacturing cheap medicines and vaccines which are needed in the world’s poorest countries.

Juncos profit is estimated to decrease by a lot in the near future, but according to the United Nations about 280 more lives are saved yearly because Juncos changed their goals.

### High amount – Helps indirectly

The pharmaceutical company Juncos have specialized in manufacturing an effective libido-increasing supplement. Many talented scientists work at Juncos, and for many years they have profited over 5 million dollars yearly.

Recently the chair members at Juncos, with the support of the stakeholders, decided that the company should strive to not only profit but also help others. Therefore Juncos started donating 50 % percent of their yearly profits from the effective libido-increasing supplement to existing charity organizations who manufacture and distribute medicines and vaccines which are need in the world’s poorest countries.

Juncos are expected to continue profiting largely yearly, but according to the United Nations about 1400 more lives are saved yearly because of Junco’s donations.

### Low amount – Helps indirectly

The pharmaceutical company Juncos have specialized in manufacturing an effective libido-increasing supplement. Many talented scientists work at Juncos, and for many years they have profited over 5 million dollars yearly.

Recently the chair members at Juncos, with the support of the stakeholders, decided that the company should strive to not only profit but also help others. Therefore Juncos started donating 50 % percent of their yearly profits from the effective libido-increasing supplement to existing charity organizations who manufacture and distribute medicines and vaccines which are need in the world’s poorest countries.

Juncos are expected to continue profiting largely yearly, but according to the United Nations about 280 more lives are saved yearly because of Junco’s donations.

## Keeping help private vignette: Individual

### High amount – Keeps helping private

Susan is 58 years old and is employed as senior manager at a big department store. She lives alone in a house that she owns without any mortgage. She also has some savings which she is planning to use for traveling abroad in the future.

A week ago, Susan saw a news segment in which a little girl with late-stage cancer was interviewed.

The little girl talked about her condition, her dreams and her fear of death. After watching the news segment, Susan decided to donate money to the Child Cancer Foundation. The day after the news segment, Susan made a one-time donation of 250 dollars.

Because Susan donated a larger amount money, she was given a thank-you letter and certificate from the Child Cancer Foundation. Susan put the letter and certificate in one of her drawers and soon forgot about it.

### Low amount – Keeps helping private

Susan is 58 years old and is employed as senior manager at a big department store. She lives alone in a house that she owns without any mortgage. She also has some savings which she is planning to use for traveling abroad in the future.

A week ago, Susan saw a news segment in which a little girl with late-stage cancer was interviewed.

The little girl talked about her condition, her dreams and her fear of death. After watching the news segment, Susan decided to donate money to the Child Cancer Foundation. The day after the news segment, Susan made a one-time donation of 50 dollars.

Because Susan donated a larger amount money, she was given a thank-you letter and certificate from the Child Cancer Foundation. Susan put the letter and certificate in one of her drawers and soon forgot about it.

### High amount – Makes helping public

Susan is 58 years old and is employed as senior manager at a big department store. She lives alone in a house that she owns without any mortgage. She also has some savings which she is planning to use for traveling abroad in the future.

A week ago, Susan saw a news segment in which a little girl with late-stage cancer was interviewed.

The little girl talked about her condition, her dreams and her fear of death. After watching the news segment, Susan decided to donate money to the Child Cancer Foundation. The day after the news segment, Susan made a one-time donation of 250 dollars.

Because Susan donated a larger amount money, she was given a thank-you letter and certificate from the Child Cancer Foundation. Susan framed the letter and certificate and hung it outside her office.

### Low amount – Makes helping public

Susan is 58 years old and is employed as senior manager at a big department store. She lives alone in a house that she owns without any mortgage. She also has some savings which she is planning to use for traveling abroad in the future.

A week ago, Susan saw a news segment in which a little girl with late-stage cancer was interviewed.

The little girl talked about her condition, her dreams and her fear of death. After watching the news segment, Susan decided to donate money to the Child Cancer Foundation. The day after the news segment, Susan made a one-time donation of 50 dollars.

Because Susan donated a larger amount money, she was given a thank-you letter and certificate from the Child Cancer Foundation. Susan framed the letter and certificate and hung it outside her office.

## Keeping help private vignette: Corporation

### High amount – Keeps helping private

The chain store Kaleido specializes in smartphone accessories and has in total 13 stores around Sweden. Kaleido has an annual turnover of 2.5 million dollars.

The latest fundraiser gala was held to help children and teens suffering from Leukemia. Before the fundraiser galas take place, the TV stations who host them usually contact companies who have donated before. Fairly recently, Kaleido was contacted and asked if they wanted to donate to this year’s fundraising gala.

This year, Kaleido decided to be one of the “big sponsors” for the yearly fundraising gala, which entails donating 5 000 dollars.

Everybody who is a sponsor may decide if their donation shall be made publicly or anonymously during the fundraising gala. Kaleido wanted to donate anonymously.

### Low amount – Keeps helping private

The chain store Kaleido specializes in smartphone accessories and has in total 13 stores around Sweden. Kaleido has an annual turnover of 2.5 million dollars.

The latest fundraiser gala was held to help children and teens suffering from Leukemia. Before the fundraiser galas take place, the TV stations who host them usually contact companies who have donated before. Fairly recently, Kaleido was contacted and asked if they wanted to donate to this year’s fundraising gala.

This year, Kaleido decided to be one of the “big sponsors” for the yearly fundraising gala, which entails donating 1 000 dollars.

Everybody who is a sponsor may decide if their donation shall be made publicly or anonymously during the fundraising gala. Kaleido wanted to donate anonymously.

### High amount – Makes helping public

The chain store Kaleido specializes in smartphone accessories and has in total 13 stores around Sweden. Kaleido has an annual turnover of 2.5 million dollars.

The latest fundraiser gala was held to help children and teens suffering from Leukemia. Before the fundraiser galas take place, the TV stations who host them usually contact companies who have donated before. Fairly recently, Kaleido was contacted and asked if they wanted to donate to this year’s fundraising gala.

This year, Kaleido decided to be one of the “big sponsors” for the yearly fundraising gala, which entails donating 5 000 dollars.

Everybody who is a sponsor may decide if their donation shall be made publicly or anonymously during the fundraising gala. Kaleido wanted their donation to be presented clearly and publicly during the fundraising gala.

### Low amount – Makes helping public

The chain store Kaleido specializes in smartphone accessories and has in total 13 stores around Sweden. Kaleido has an annual turnover of 2.5 million dollars.

The latest fundraiser gala was held to help children and teens suffering from Leukemia. Before the fundraiser galas take place, the TV stations who host them usually contact companies who have donated before. Fairly recently, Kaleido was contacted and asked if they wanted to donate to this year’s fundraising gala.

This year, Kaleido decided to be one of the “big sponsors” for the yearly fundraising gala, which entails donating 1 000 dollars.

Everybody who is a sponsor may decide if their donation shall be made publicly or anonymously during the fundraising gala. Kaleido wanted their donation to be presented clearly and publicly during the fundraising gala.

## Matching other’s donation vignette: Individual

### High amount – Matching other

Anna and her acquaintance Rebecca are out on a business lunch when they are approached by a volunteer working for a reputable charity organization. The volunteer asks if they could potentially donate a small amount towards helping a local cat shelter.

The acquaintance Rebecca immediately agrees and donates 20 dollars.

Anna notices the amount Rebecca donated and then also donates 20 dollars.

### Low amount – Matching other

Anna and her acquaintance Rebecca are out on a business lunch when they are approached by a volunteer working for a reputable charity organization. The volunteer asks if they could potentially donate a small amount towards helping a local cat shelter.

The acquaintance Rebecca immediately agrees and donates 4 dollars.

Anna notices the amount Rebecca donated and then also donates 4 dollars.

### High amount – Surpassing other

Anna and her acquaintance Rebecca are out on a business lunch when they are approached by a volunteer working for a reputable charity organization. The volunteer asks if they could potentially donate a small amount towards helping a local cat shelter.

The acquaintance Rebecca immediately agrees and donates 10 dollars.

Anna notices the amount Rebecca donated and then donates 20 dollars.

### Low amount – Surpassing other

Anna and her acquaintance Rebecca are out on a business lunch when they are approached by a volunteer working for a reputable charity organization. The volunteer asks if they could potentially donate a small amount towards helping a local cat shelter.

The acquaintance Rebecca immediately agrees and donates 2 dollars.

Anna notices the amount Rebecca donated and then donates 4 dollars.

## Matching other’s donation vignette: Corporation

### High amount – Matching other

The companies Amos and Betos are equally large and operate in the same average-sized town. Both Amos and Betos are contacted one day by a foundation which cares for homeless dogs. The foundation asks for a contribution which will be used towards building a new dog shelter in the town.

Betos immediately decided to donate 2 000 dollars to towards the shelter.

After Amos has heard of Betos’ donation, they decide to donate 2 000 dollars as well.

### Low amount – Matching other

The companies Amos and Betos are equally large and operate in the same average-sized town. Both Amos and Betos are contacted one day by a foundation which cares for homeless dogs. The foundation asks for a contribution which will be used towards building a new dog shelter in the town.

Betos immediately decided to donate 400 dollars to towards the shelter.

After Amos has heard of Betos’ donation, they decide to donate 400 dollars as well.

### High amount – Surpassing other

The companies Amos and Betos are equally large and operate in the same average-sized town. Both Amos and Betos are contacted one day by a foundation which cares for homeless dogs. The foundation asks for a contribution which will be used towards building a new dog shelter in the town.

Betos immediately decided to donate 1 000 dollars to towards the shelter.

After Amos has heard of Betos’ donation, they decide to donate 2 000 dollars.

### Low amount – Surpassing other

The companies Amos and Betos are equally large and operate in the same average-sized town. Both Amos and Betos are contacted one day by a foundation which cares for homeless dogs. The foundation asks for a contribution which will be used towards building a new dog shelter in the town.

Betos immediately decided to donate 200 dollars to towards the shelter.

After Amos has heard of Betos’ donation, they decide to donate 400 dollars.

## Equal helping vignette: Individual

### High amount – Gives to all requesters

John is a 45-year-old blue-collar worker. Recently John won 1 million dollars in a state-run lottery. When people win big amounts in lotteries, different charitable organizations usually contact the winner to ask if they potentially could donate a part of the sum to their charity.

John got contacted by 12 different, respected charitable organizations who specialize in different kinds of aid for those in need. John agreed to donate to all of the 12 charitable organizations who contacted him. He chose to donate equal amounts to each of the organizations.

In total, John donated 30 000 dollars to the 12 charitable organizations.

### Low amount – Gives to all requesters

John is a 45-year-old blue-collar worker. Recently John won 1 million dollars in a state-run lottery. When people win big amounts in lotteries, different charitable organizations usually contact the winner to ask if they potentially could donate a part of the sum to their charity.

John got contacted by 12 different, respected charitable organizations who specialize in different kinds of aid for those in need. John agreed to donate to all of the 12 charitable organizations who contacted him. He chose to donate equal amounts to each of the organizations.

In total, John donated 5 000 dollars to the 12 charitable organizations.

### High amount – Gives to one requester

John is a 45-year-old blue-collar worker. Recently John won 1 million dollars in a state-run lottery. When people win big amounts in lotteries, different charitable organizations usually contact the winner to ask if they potentially could donate a part of the sum to their charity.

John got contacted by 12 different, respected charitable organizations who specialize in different kinds of aid for those in need. John declined to donate to 11 of the 12 charitable organizations who contacted him. He only agreed to donate towards the one charitable organization he got the best impression of.

In total, John donated 30 000 dollars to that charitable organization.

### Low amount – Gives to one requester

John is a 45-year-old blue-collar worker. Recently John won 1 million dollars in a state-run lottery. When people win big amounts in lotteries, different charitable organizations usually contact the winner to ask if they potentially could donate a part of the sum to their charity.

John got contacted by 12 different, respected charitable organizations who specialize in different kinds of aid for those in need. John declined to donate to 11 of the 12 charitable organizations who contacted him. He only agreed to donate towards the one charitable organization he got the best impression of.

In total, John donated 5 000 dollars to that charitable organization.

## Equal helping vignette corporation

### High amount – Gives to all requesters

Lopin is a bank in an averaged sized city which this year have profited 2.5 million dollars.

Every year banks are asked by different organizations if they could potentially sponsor various local projects which aim to increase the local inhabitant’s well-being. This year, eight serious and realistic proposals for projects where put forward to Lopin from different organizations located in the city.

During their last board meeting, Lupin decided to accept to sponsor all eight projects this year.

In total Lupin donated 200 000 dollars distributed equally to all the projects.

### Low amount – Gives to all requesters

Lopin is a bank in an averaged sized city which this year have profited 2.5 million dollars.

Every year banks are asked by different organizations if they could potentially sponsor various local projects which aim to increase the local inhabitant’s well-being. This year, eight serious and realistic proposals for projects where put forward to Lopin from different organizations located in the city.

During their last board meeting, Lupin decided to accept to sponsor all eight projects this year.

In total Lupin donated 40 000 dollars distributed equally to all the projects.

### High amount – Gives to one requester

Lopin is a bank in an averaged sized city which this year have profited 2.5 million dollars.

Every year banks are asked by different organizations if they could potentially sponsor various local projects which aim to increase the local inhabitant’s well-being. This year, eight serious and realistic proposals for projects where put forward to Lopin from different organizations located in the city.

During their last board meeting, Lupin decided to only sponsor one project this year. The other seven projects where left unfinanced.

In total Lopin donated 200 000 dollars to the local project the experts had deemed as the best.

### Low amount – Gives to one requester

Lopin is a bank in an averaged sized city which this year have profited 2.5 million dollars.

Every year banks are asked by different organizations if they could potentially sponsor various local projects which aim to increase the local inhabitant’s well-being. This year, eight serious and realistic proposals for projects where put forward to Lopin from different organizations located in the city.

During their last board meeting, Lupin decided to only sponsor one project this year. The other seven projects where left unfinanced.

In total Lopin donated 40 00 dollars to the local project the experts had deemed as the best.
