# Supplementary material for: Type and amount of help as predictors for impression of helpers
Source: PLoS One. 2020 Dec 11;15(12):e0243808. doi: 10.1371/journal.pone.0243808 (PMC7732071; doi:10.1371/journal.pone.0243808)
Supplement: S5 File — (F-statistics and significance-values). (DOCX) [file pone.0243808.s005.docx]

# Online supplementary material (OSM) 5. Main effects and interaction effects in Study 2.

Table S5: Results from the 2×2×2 ANOVAS (main effects and interaction effect) from all vignettes included in Study 1a and 1b. The three-way interaction was non-significant in all vignettes. The significant interaction effects are interpreted below

|  | **Study 2** | | | | | |
| --- | --- | --- | --- | --- | --- | --- |
|  | ***Type of help main effect*** | ***Amount of help main effect*** | ***Utility reminder main effect*** | ***Type*Amount  effect*** | ***Utility reminder* Type effect*** | ***Utility reminder* Amount effect*** |
| Directness-  Individual | *F*[1, 390] = 8.21,  *p* = .004, *η*_p_^2^ = .021** | *F*[1, 390] = 3.77,  *p* = .053, *η*_p_^2^ = .010† | *F*[1, 390] = 7.74,  *p* = .006, *η*_p_^2^ = .019** | *F*[1, 390] = 4.00,  *p* = .046.* | *F*[1, 390] = 1.04,  *p* = .309. | *F*[1, 390] = 0.99,  *p* = .321. |
| Directness- Corporation | *F*[1, 390] = 27.08,  *p* < .001, *η*_p_^2^ = .065*** | *F*[1, 390] = 1.24,  *p* = .265, *η*_p_^2^ = .003 | *F*[1, 390] = 6.55,  *p* = .011, *η*_p_^2^ = .017* | *F*[1, 390] = 2.47,  *p* = .117. | *F*[1, 390] = 0.32,  *p* = .571. | *F*[1, 390] = 0.05,  *p* = .816. |
| Keeping help private- Individual | *F*[1, 390] = 6.30,  *p* = .012, *η*_p_^2^ = .016* | *F*[1, 390] = 1.08,  *p* = .298, *η*_p_^2^ = .003 | *F*[1, 390] = 2.06,  *p* = .152, *η*_p_^2^ = .005 | *F*[1, 390] = 10.09,  *p* = .002.** | *F*[1, 390] = 1.01,  *p* = .315. | *F*[1, 390] = 4.48,  *p* = .035.* |
| Keeping help private- Corporation | *F*[1, 389] = 246.06,  *p* < .001, *η*_p_^2^ = .387*** | *F*[1, 389] = 25.20,  *p* < .001, *η*_p_^2^ = .061* | *F*[1, 389] = 6.10,  *p* = .014, *η*_p_^2^ = .015* | *F*[1, 389] = 0.02,  *p* = .876 | *F*[1, 389] = 0.91,  *p* = .340 | *F*[1, 389] = 1.29,  *p* = .256 |
| Matching others – Individual | *F*[1, 389] = 23.32,  *p* < .001, *η*_p_^2^ = .057*** | *F*[1, 389] = 0.31,  *p* = .581, *η*_p_^2^ = .001 | *F*[1, 389] = 3.18,  *p* = .075, *η*_p_^2^ = .008† | *F*[1, 389] = 10.76,  *p* = .001** | *F*[1, 389] = 0.22,  *p* = .642 | *F*[1, 389] = 0.74,  *p* = .390 |
| Matching others – Corporation | *F*[1, 390] = 1.18,  *p* = .278, *η*_p_^2^ = .003 | *F*[1, 390] = 1.26,  *p* = .263, *η*_p_^2^ = .003 | *F*[1, 390] = 14.40,  *p* < .001, *η*_p_^2^ = .036*** | *F*[1, 390] = 0.24,  *p* = .626 | *F*[1, 390] = 4.77,  *p* = .030* | *F*[1, 390] = 0.15,  *p* = .696 |
| Equal helping – Individual | *F*[1, 389] = 14.32,  *p* < .001, *η*_p_^2^ = .036*** | *F*[1, 389] = 13.62,  *p* < .001, *η*_p_^2^ = .034*** | *F*[1, 389] = 3.81,  *p* = .052, *η*_p_^2^ = .010† | *F*[1, 389] = 4.48,  *p* = .035* | *F*[1, 389] = 0.44,  *p* = .509 | *F*[1, 389] = 0.19,  *p* = .662 |
| Equal helping- Corporation | *F*[1, 390] = 56.80,  *p* < .001, *η*_p_^2^ = .127*** | *F*[1, 390] = 2.89,  *p* = .090, *η*_p_^2^ = .007† | *F*[1, 390] = 19.26,  *p* < .001, *η*_p_^2^ = .047*** | *F*[1, 390] = 1.14,  *p* = .287 | *F*[1, 390] = 1.01,  *p* = .316 | *F*[1, 390] = 0.85,  *p* = .358 |

*** = p <.001, ** = p < .01, * = p < .05, † = p < .1

## Directness vignette: Individual

Type × amount of help interaction *η*_p_^2^ = .01 [.00 − .03]. This interaction should be understood as a higher amount of help slightly improving impressions when the help is done indirectly but not when the help is done directly.

## Keeping help private vignette: Individual

Type × amount of help interaction *η*_p_^2^ = .03[.01 − .06]. This interaction should be understood as amount of help slightly improving impressions when helping is made public, but as slightly deteriorating impressions when helping is kept private.

Utility-reminder × amount of help interaction *η*_p_^2^ = .01[.00 − .03] Amount of help improved impressions of helpers for participants exposed to the utility-prime but not for participants exposed to the control question.

## Matching other’s donation vignette: Individual

Type × amount of help interaction *η*_p_^2^ = .03 [.01 − .06] This interaction should be understood as a higher amount of help slightly improving impressions when matching another person’s donation but slightly deteriorating impressions when surpassing another person’s donation.

## Matching other’s donation vignette: Corporation

Utility-reminder × type of help interaction *η*_p_^2^ = .01 [.00 − .04]. This can be understood as the utility-prime improving impressions of organizations who surpassed another organization’s donation but not as much for an organization who matched another donation.

## Equal helping vignette: Individual

Type × amount of help interaction *η*_p_^2^ = .01 [.00 − .03] This can be understood as the amount of help positively predicting impressions when the helper donates to a single organization but not when she donates to all organizations.
