# Supplementary material for: Type and amount of help as predictors for impression of helpers
Source: PLoS One. 2020 Dec 11;15(12):e0243808. doi: 10.1371/journal.pone.0243808 (PMC7732071; doi:10.1371/journal.pone.0243808)

**Online supplementary material (OSM) 6:** **Graphical illustrations of the results from each vignette in all studies**


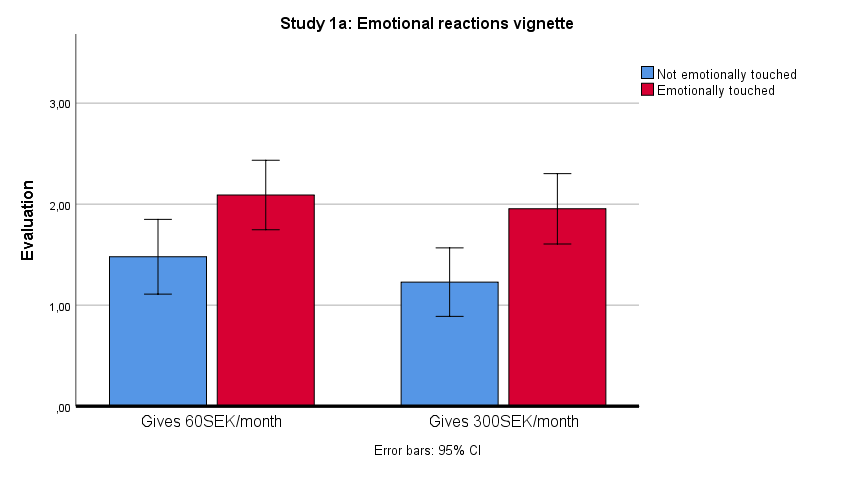

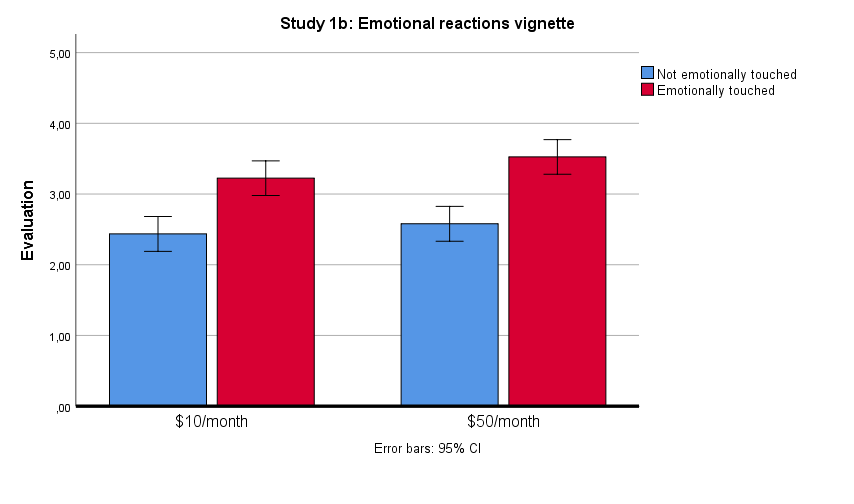


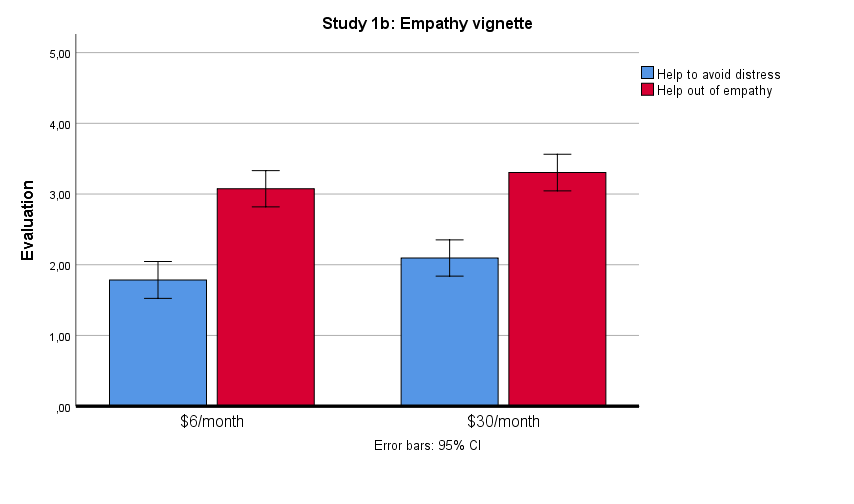


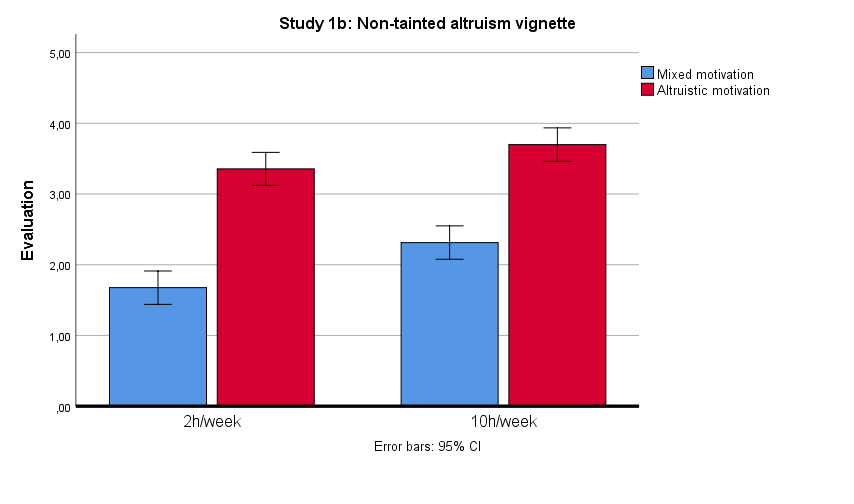


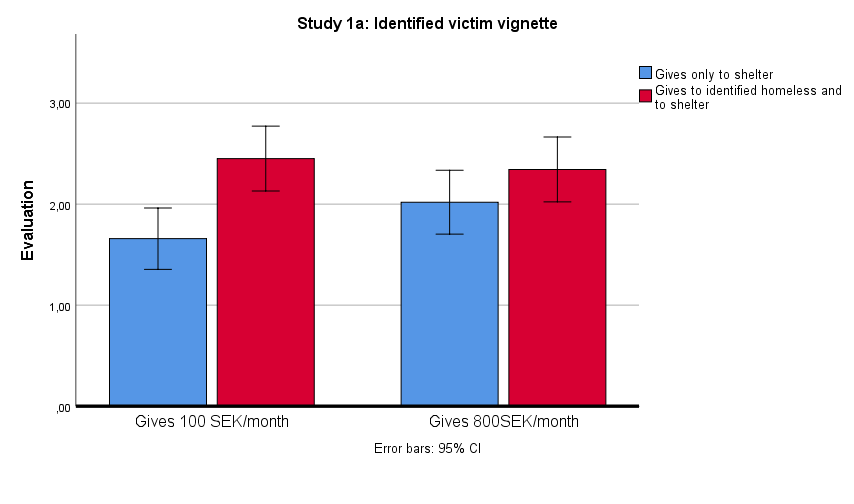

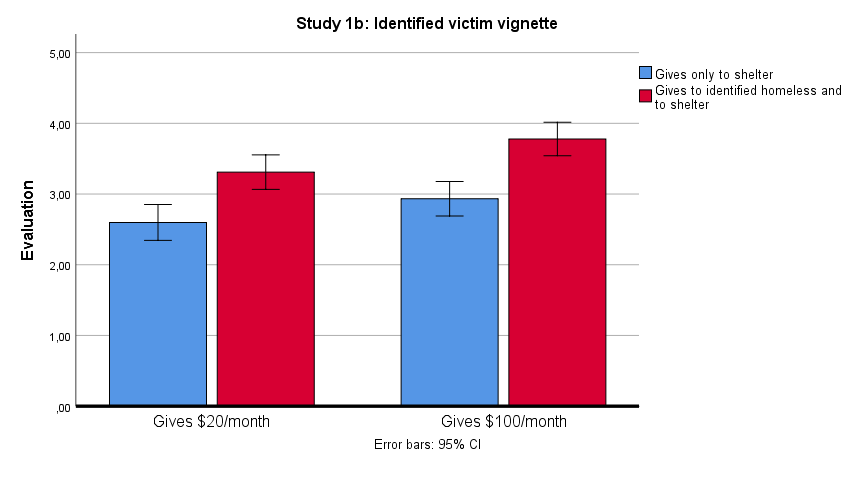


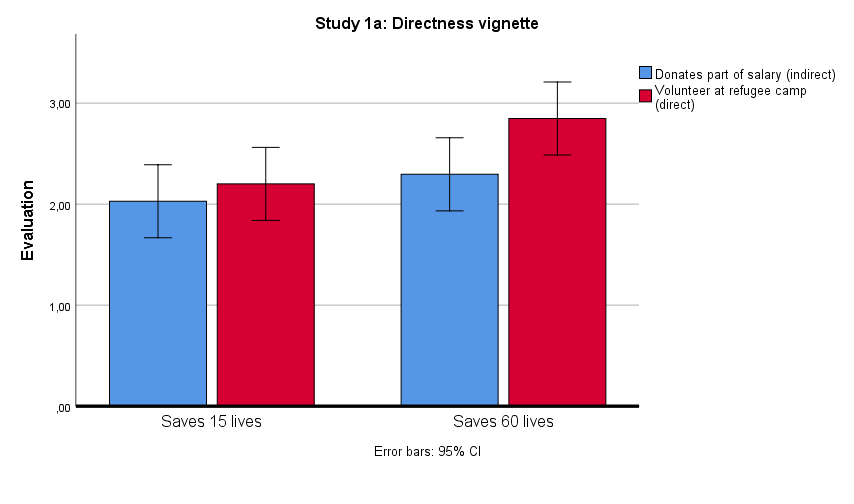

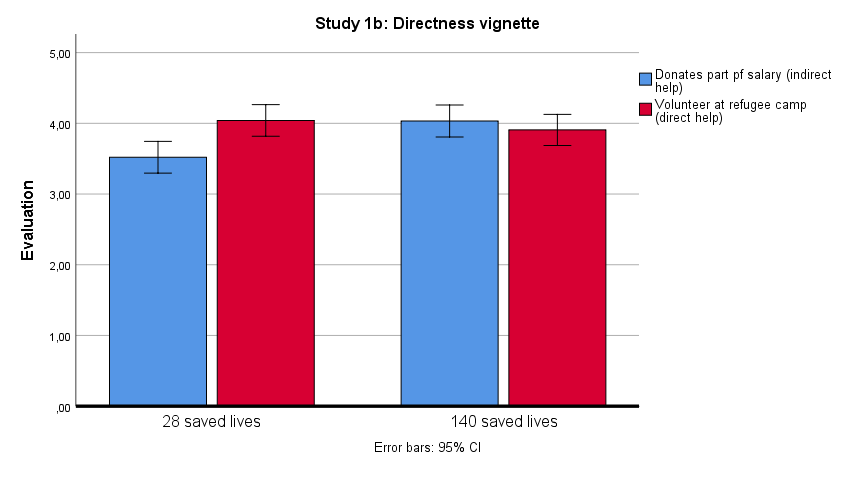


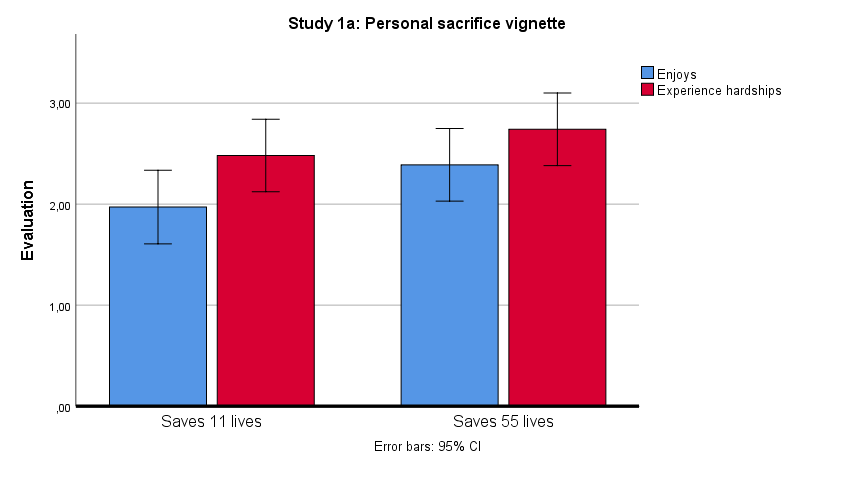

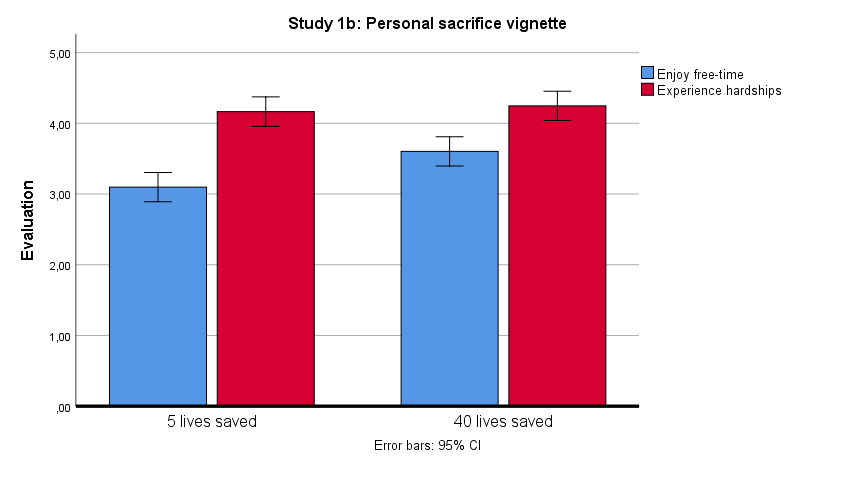


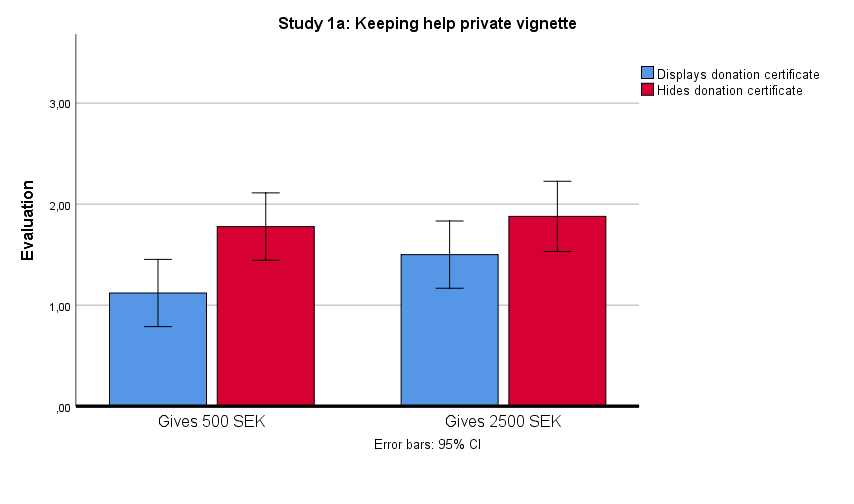

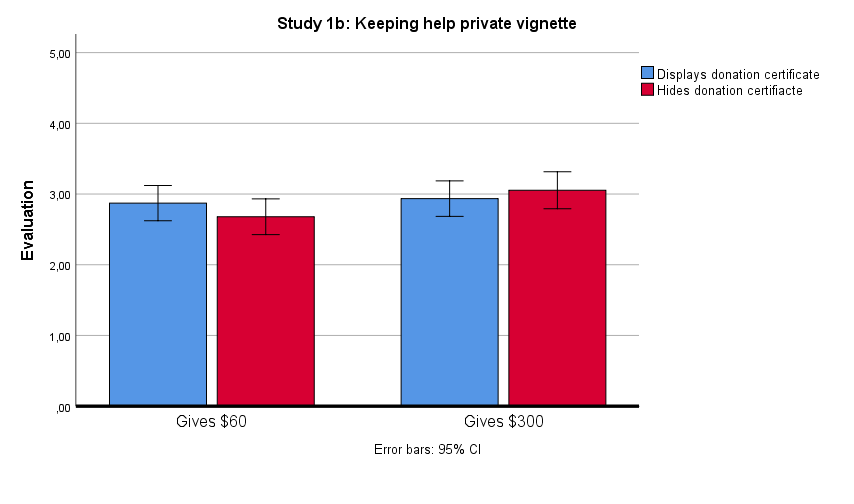


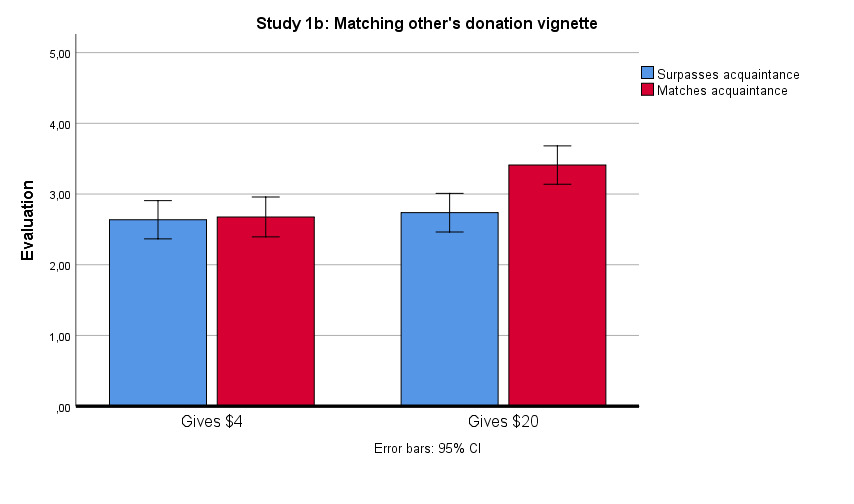


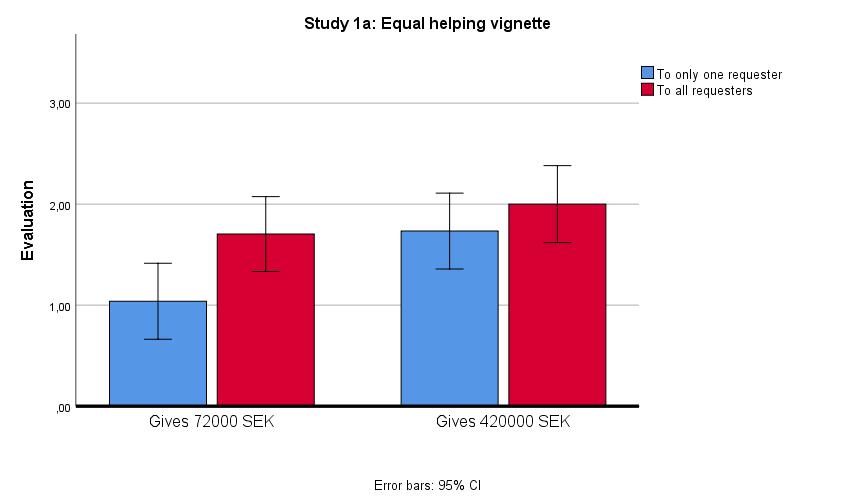

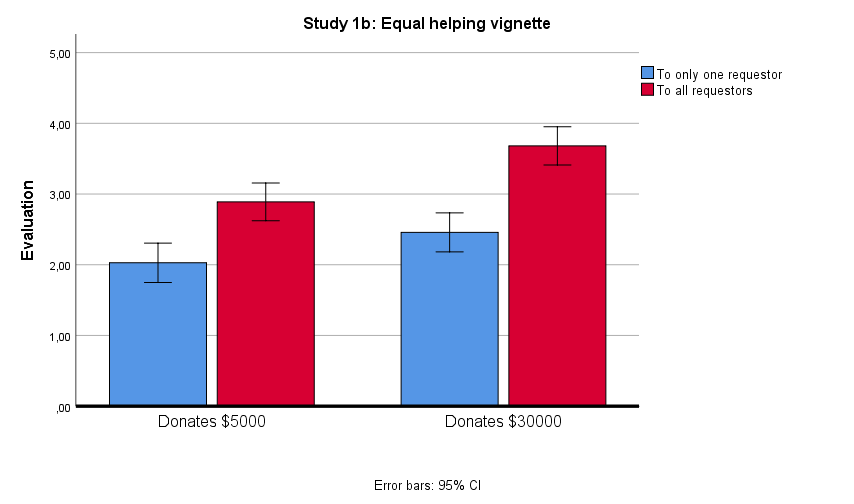


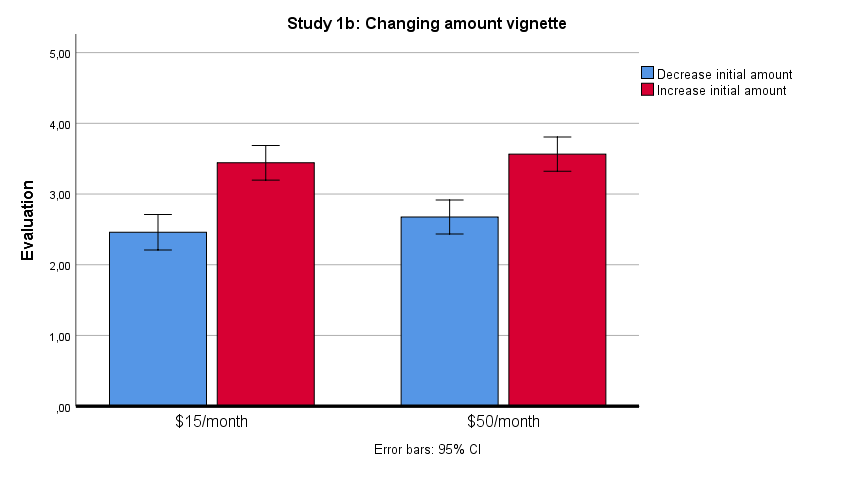


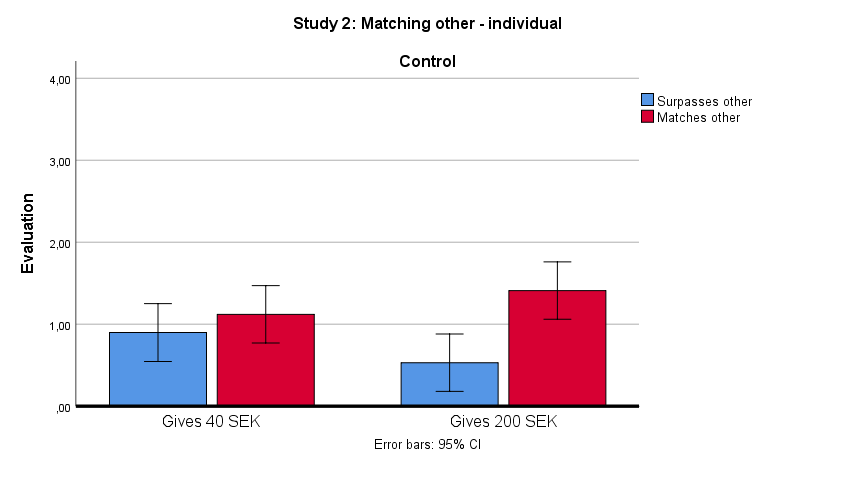

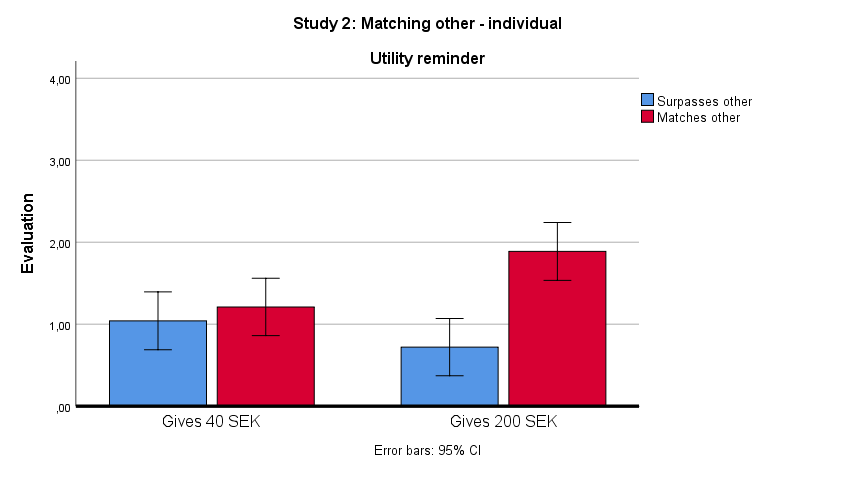


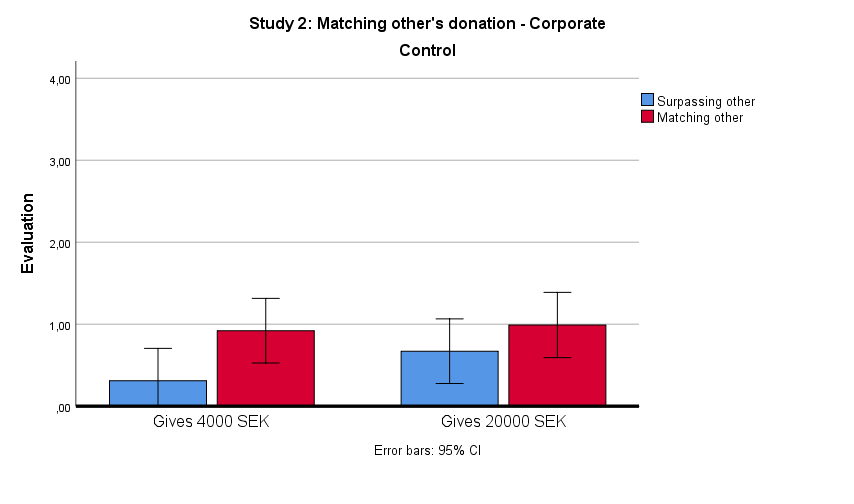

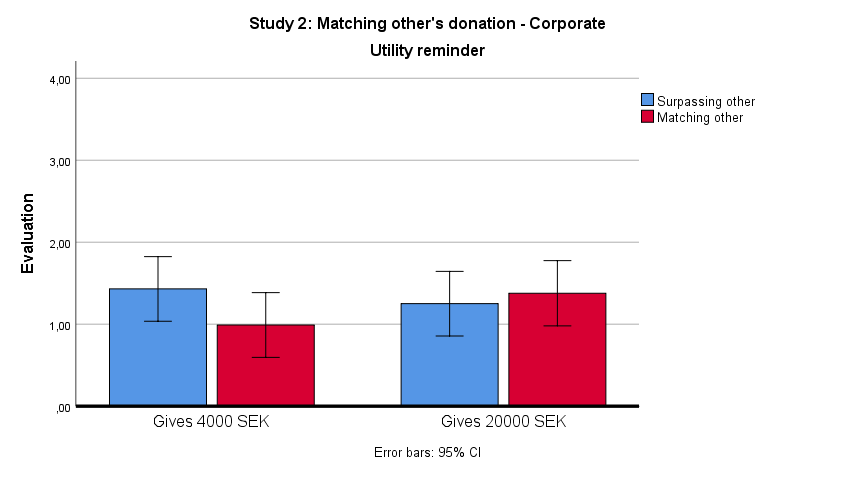


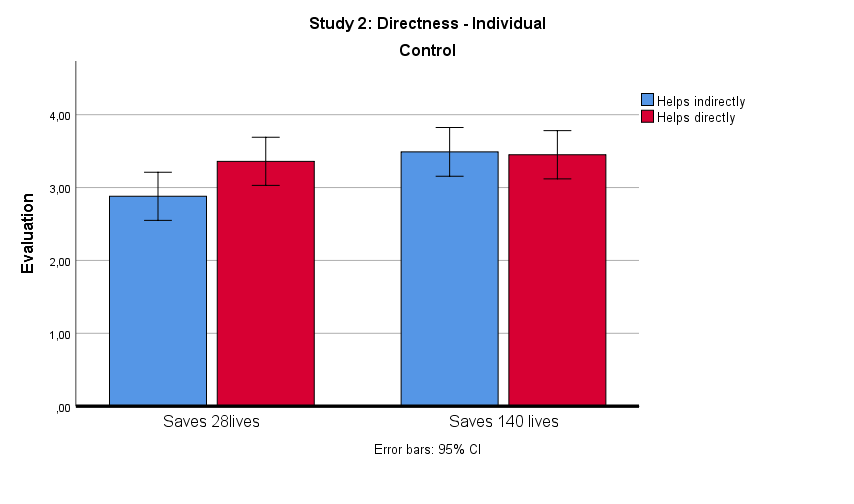

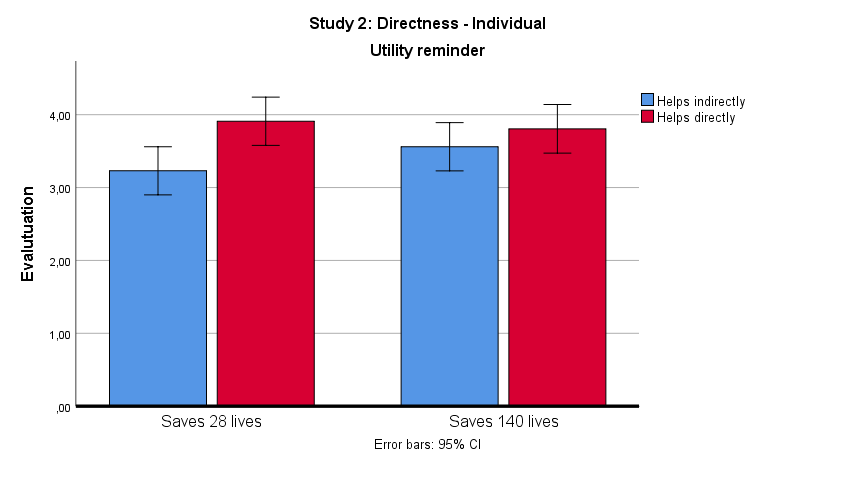


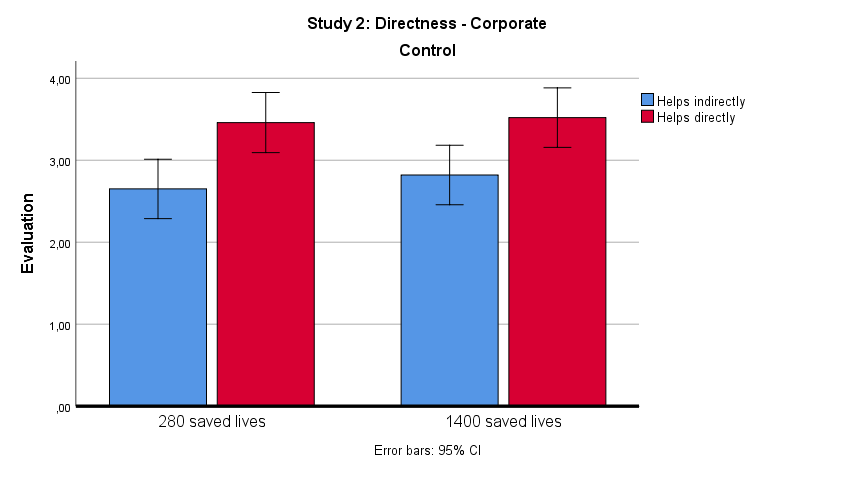

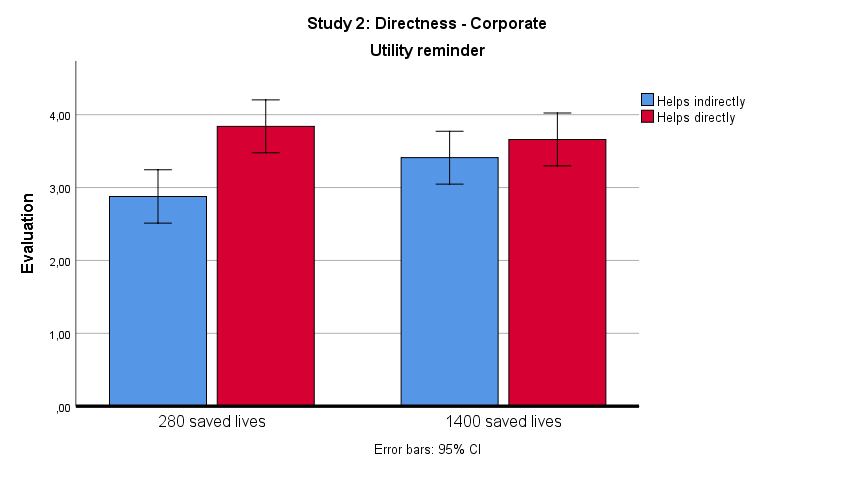


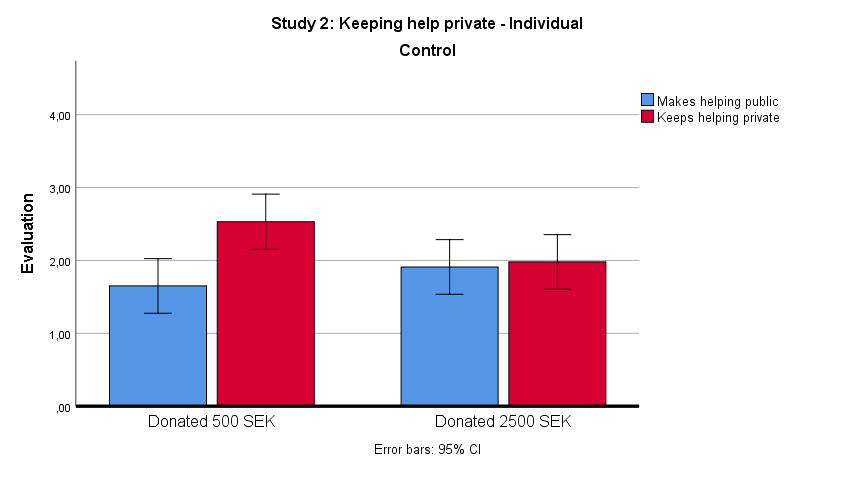

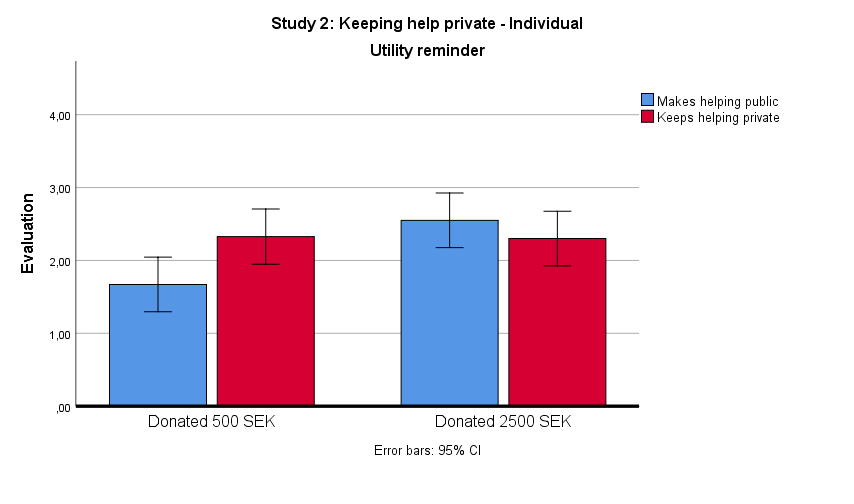


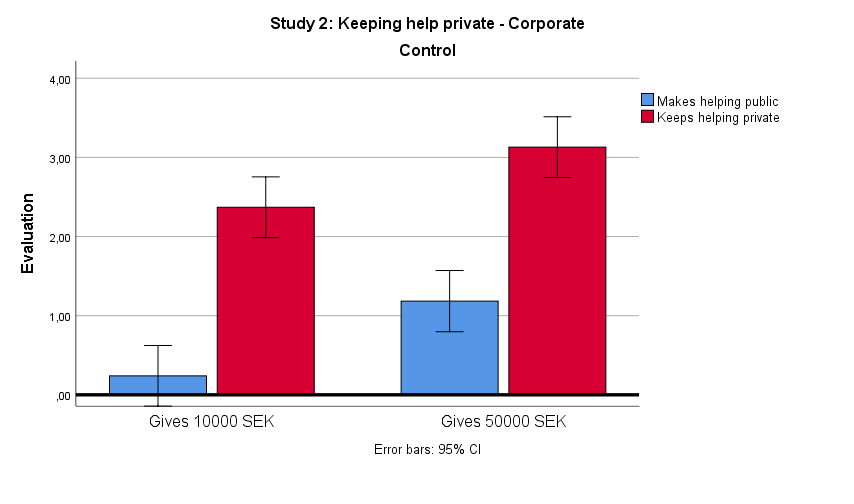

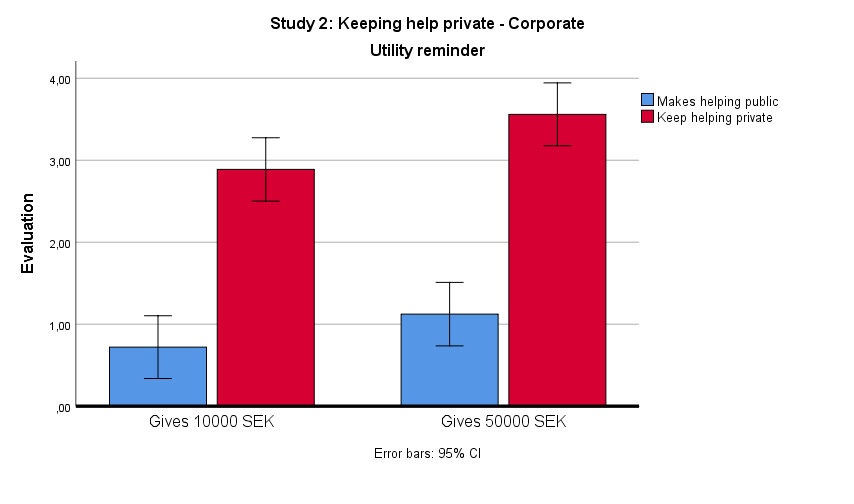


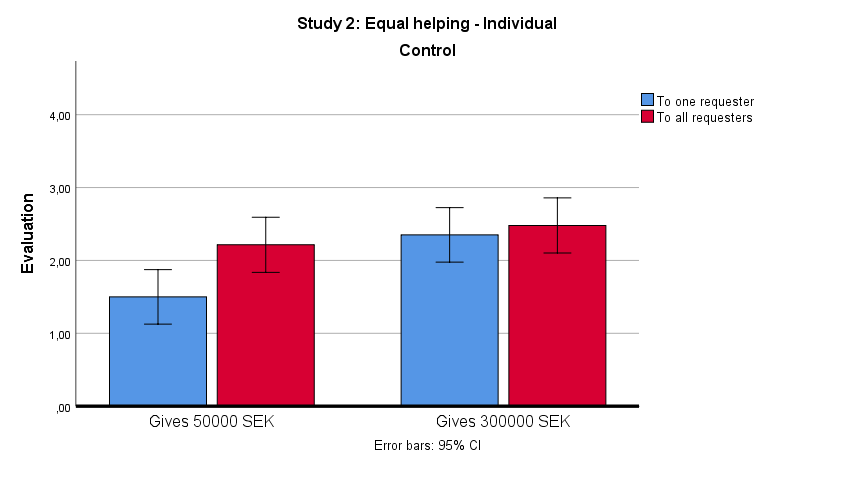

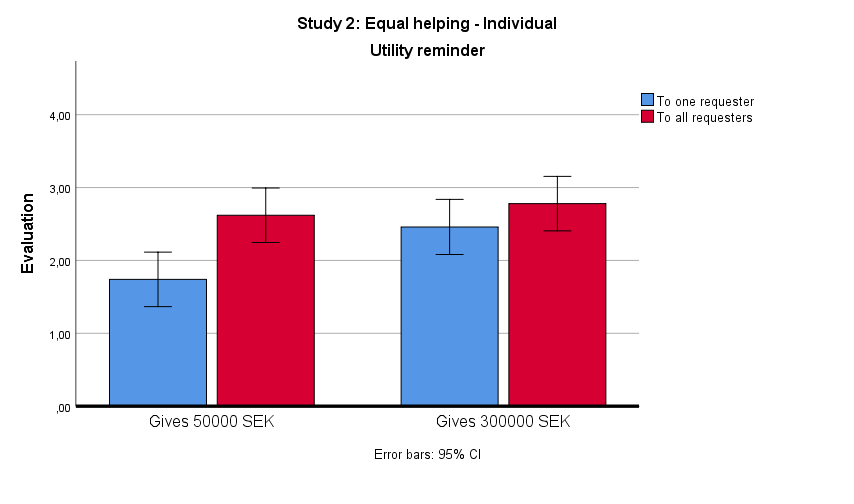


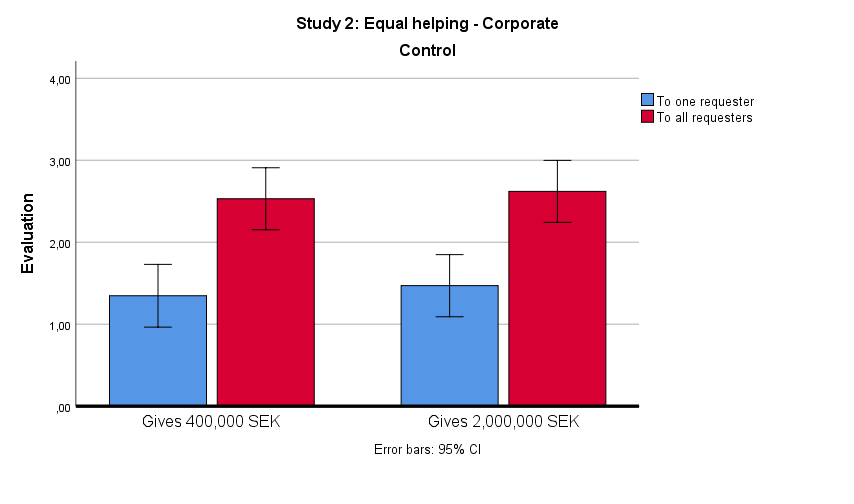

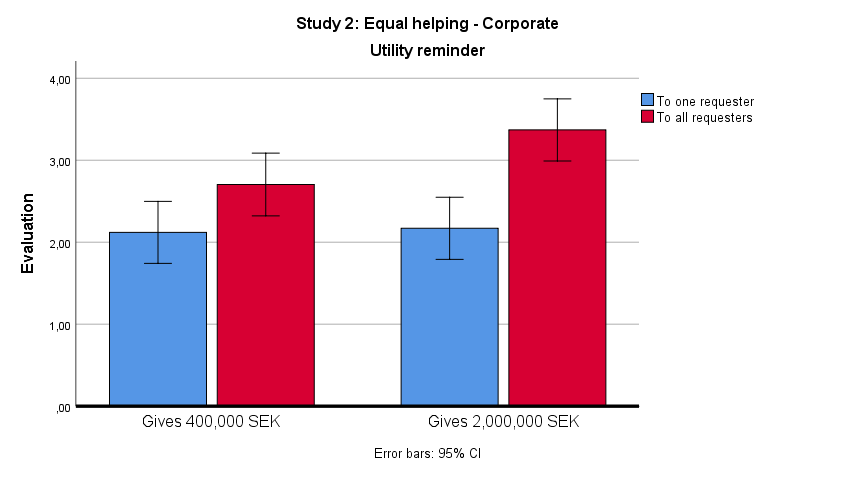

Supplement: S6 File — (DOCX) [file pone.0243808.s006.docx]
